# Multimodal host–guest complexation for efficient and stable perovskite photovoltaics

**DOI:** 10.1038/s41467-021-23566-2

**Published:** 2021-06-07

**Authors:** Hong Zhang, Felix Thomas Eickemeyer, Zhiwen Zhou, Marko Mladenović, Farzaneh Jahanbakhshi, Lena Merten, Alexander Hinderhofer, Michael A. Hope, Olivier Ouellette, Aditya Mishra, Paramvir Ahlawat, Dan Ren, Tzu-Sen Su, Anurag Krishna, Zaiwei Wang, Zhaowen Dong, Jinming Guo, Shaik M. Zakeeruddin, Frank Schreiber, Anders Hagfeldt, Lyndon Emsley, Ursula Rothlisberger, Jovana V. Milić, Michael Grätzel

**Affiliations:** 1 Laboratory of Photonics and Interfaces, Institute of Chemical Sciences and Engineering, École Polytechnique Fédérale de Lausanne, Lausanne, Switzerland; 2 Laboratory of Computational Chemistry and Biochemistry, Institute of Chemical Sciences and Engineering, École Polytechnique Fédérale de Lausanne, Lausanne, Switzerland; 3 Institut für Angewandte Physik, Universität Tübingen, Tübingen, Germany; 4 Laboratory of Magnetic Resonance, Institute of Chemical Sciences and Engineering, École Polytechnique Fédérale de Lausanne, Lausanne, Switzerland; 5 Laboratory of Photomolecular Science, Institute of Chemical Sciences and Engineering, École Polytechnique Fédérale de Lausanne, Lausanne, Switzerland; 6 Laboratory of Supramolecular Chemistry, Institute of Chemical Sciences and Engineering, École Polytechnique Fédérale de Lausanne, Lausanne, Switzerland; 7 Laboratory for Biological Geochemistry, École Polytechnique Fédérale de Lausanne, Lausanne, Switzerland; 8 Adolphe Merkle Institute of the University of Fribourg in Switzerland, Fribourg, Switzerland; 9 Present Address: Department of Chemistry – Ångström Laboratory, Uppsala University, Uppsala, Sweden

**Keywords:** Supramolecular chemistry, Energy harvesting, Materials for energy and catalysis

## Abstract

Formamidinium lead iodide perovskites are promising light-harvesting materials, yet stabilizing them under operating conditions without compromising optimal optoelectronic properties remains challenging. We report a multimodal host–guest complexation strategy to overcome this challenge using a crown ether, dibenzo-21-crown-7, which acts as a vehicle that assembles at the interface and delivers Cs^+^ ions into the interior while modulating the material. This provides a local gradient of doping at the nanoscale that assists in photoinduced charge separation while passivating surface and bulk defects, stabilizing the perovskite phase through a synergistic effect of the host, guest, and host–guest complex. The resulting solar cells show power conversion efficiencies exceeding 24% and enhanced operational stability, maintaining over 95% of their performance without encapsulation for 500 h under continuous operation. Moreover, the host contributes to binding lead ions, reducing their environmental impact. This supramolecular strategy illustrates the broad implications of host–guest chemistry in photovoltaics.

## Abstract (web summary)

It remains a challenge to achieve a balance between performance and stability, as well as addressing the environmental impact of perovskite solar cells. Here, the authors propose a multimodal host-guest complexation strategy enabling these shortcomings to be addressed simultaneously.

## Introduction

Perovskite solar cells (PSCs) presently attain high power conversion efficiencies (PCE) and show the potential for low-cost fabrication, positioning them as one of the leading candidates for the next generation of thin-film photovoltaics^1–6^. They nevertheless still suffer from poor operational stability and degradation under ambient conditions, while presenting a potential negative environmental impact from the toxic lead component^6^. Moreover, the performance is still limited by defects and impurities that enhance non-radiative recombination of photogenerated charge carriers^7–12^. Conventional passivation is an effective strategy to remove defects from the surface of films^8,13–15^, yet a number of defects remain in the bulk and it is vital to mitigate both types of defects. Formamidinium lead iodide (FAPbI_3_) and FAPbI_3_-rich perovskites are particularly preferred for photovoltaic applications due to their superior optoelectronic properties and thermal stability^5^. However, the photoactive black phase (3C, α) readily transforms to the undesired wide-bandgap (2H, δ) phase under ambient conditions. Moreover, several polytypes (i.e., 2H, 4H, and 6H) can be formed, as evidenced both experimentally and theoretically^16^, with the δ polytype being the most thermodynamically stable at ambient temperature (Supplementary Fig. 1). To address the thermodynamic instability of α-FAPbI_3_, it has been previously shown that using alkali metal cations could be an effective strategy to stabilize the black phase^17,18^. However, this bulk approach results in homogenous doping that comes at the expense of increasing the bandgap and hampering the formation of high-quality films without addressing the detrimental lead impact^6,16–18^.

**Fig. 1:**
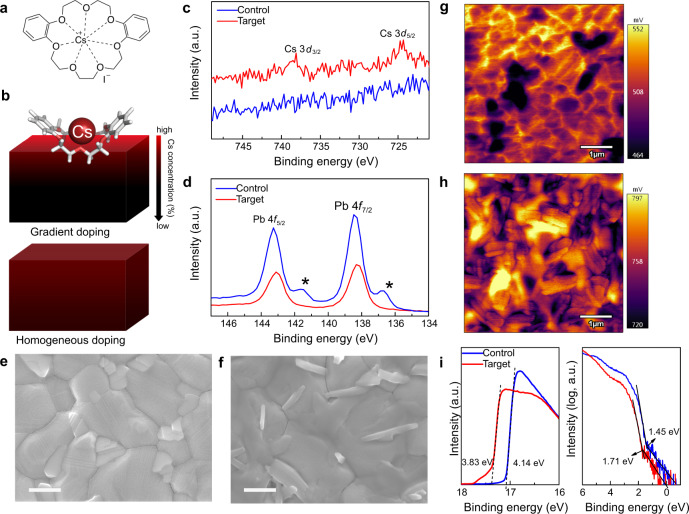
Effect of crown-ether-mediated interfacial Cs doping on the properties of perovskite films. **a** Molecular structure of the CsI–DB21C7 complex. **b** Schematic representation of the gradient and homogeneous Cs doping of perovskite films. XPS core-level spectra for Cs 3*d* (**c**) and Pb 4*f* (**d**). The asterisk (*) indicates metallic Pb species. Top-view SEM images of control (**e**) and target (**f**) perovskite films revealing surface nanostructures upon treatment. The scale bar represents 500 nm. KPFM images of control (**g**) and target (**h**) perovskite films. **i** UPS spectra of perovskite films.

Here, we introduce an unprecedented concept of multimodal host–guest-complexation of dopants to simultaneously modulate the surface and bulk composition of perovskite films through a synergistic effect of the host, guest, and the host–guest complex, which we demonstrate for the case of Cs^+^ metal ions. The polar solvents that are commonly used (e.g., water, dimethylformamide, dimethyl sulfoxide, etc.) for dissolving metal halide salts could dissolve or damage the perovskite film^17^. This prevents the use of these solvents to dissolve metal halide salts for the treatment of FAPbI_3_-based perovskites. Crown ethers are known to serve as vehicles for different ions, for example in phase-transfer catalysis^19^, forming host–guest complexes via ion–dipole interactions between the oxygen atoms of the macrocycle and the metal cation, with remarkable selectivity for certain alkali metal ions due to the complementary size^20,21^. We exploit this key property of crown ethers and their molecular assemblies to infuse Cs^+^ ions onto the perovskite film without damaging them, by dissolving the complex in an orthogonal non-polar solvent (e.g., chlorobenzene). We have employed dibenzo-21-crown-7 (DB21C7) as a proof of concept due to its strong affinity for Cs^+^ ions^20,21^, forming a well-defined host–guest complex (Fig. 1a). This complex is soluble in chlorobenzene, which is compatible with the perovskite solution-processing. This strategy was found to substantially decrease defects and improve the morphology of perovskite films without significant change in the optoelectronic properties, resulting in high performance and stability. The synergistic effect of the host, guest, and their complex enables simultaneous passivation of the surface and bulk defects, while reducing the environmental impact of lead.

## Results and discussion

### Surface modification of perovskite films

FAPbI_3_-based perovskite films were deposited on mesoporous (mp) TiO_2_ substrates via a one-step method using an antisolvent (see the Methods section). After thermal annealing, the perovskite film was treated with a solution of the CsI–DB21C7 complex. The control films were based on FAPbI_3_ or a FAPbI_3_-rich composition of (FAPbI_3_)_0.97_(MAPbBr_3_)_0.03_ unless otherwise noted. The synthesis of the CsI–DB21C7 complex is detailed in the Methods section and its complexation by cesium cations was subsequently verified by nuclear magnetic resonance (NMR; Supplementary Fig. 2). An additional annealing step was then carried out to promote the infusion of Cs^+^ into the bulk of the perovskite film, to form a gradient-doped structure, which increases stability without compromising the optimal optoelectronic properties, unlike homogeneous doping (Fig. 1b). The treatment conditions (i.e., concentration, annealing temperature, annealing duration, and counter ion) were optimized (as detailed in Supplementary Fig. 3) and samples with the optimized condition were studied further, labeled as target below.

The surface composition of the perovskite films was investigated by X-ray photoelectron spectroscopy (XPS). The XPS spectra in the Cs 3*d* level range (Fig. 1c and Supplementary Fig. 4a–d) show two signals in the treated film with binding energies of 739.0 and 725.0 eV that can be ascribed to Cs 3*d*_3/2_ and Cs 3*d*_5/2_ levels, respectively, whereas no signals are observed in the control perovskite film. This confirms that Cs^+^ has been successfully transferred to the target perovskite film. Moreover, the XPS spectra in the Pb 4*f* level range (Fig. 1d) reveal two main peaks associated with Pb 4*f*_7/2_ and Pb 4*f*_5/2_ at 138.5 and 143.3 eV, respectively, attributed to Pb–I species, along with two smaller peaks located at 136.6 and 141.6 eV that arise from the presence of metallic Pb. The metallic Pb peak vanishes in the treated film, which suggests the host crown binds to the undercoordinated Pb^2+^ ions which are responsible for the formation of metallic Pb^22^. This would greatly benefit the operational stability of the PSCs which suffer severely from the presence of elemental Pb^7–12^. Moreover, the peaks in the O 1*s* level range at 533.1 eV and C 1*s* at 286.3 eV (Supplementary Fig. 4a, b) that are associated with C–O binding energies suggest that the crown ether ligands remain on the surface of the film. In the attenuated total reflection Fourier transform infrared (ATR-FTIR) spectra (Supplementary Fig. 5), the shift of the characteristic C–O stretching vibration peaks of DB21C7 after treatment of the perovskite is consistent with the interaction between the crown ether and Pb^2+^ in the perovskite phase.

The morphology of the perovskite surface was then analyzed by scanning electron microscopy (SEM; Fig. 1e, f and Supplementary Fig. 6). The target Cs-doped perovskite film shows larger grain sizes compared to the reference, with the appearance of needle-like structures and fewer grain boundaries due to increase of the size of the grains upon treatment^22^. This could be the result of preferential binding of the DB21C7 at the boundaries, similarly to other molecular modulators that contribute to reducing grain boundaries^23^. Elemental mapping of the treated perovskite films by energy-dispersive X-ray spectroscopy (EDS) further suggests that the needle-like structures contain carbon and Cs (Supplementary Fig. 6a), and are thus likely to be the CsI–DB21C7 complex, in accordance with the XPS analysis.

In addition to these structural changes, Kelvin probe force microscopy (KPFM) and ultraviolet photoelectron spectroscopy (UPS) demonstrate that the electronic structure at the surface of the treated perovskite film differs from that of the control samples. The target Cs-doped perovskite surface exhibited a higher and more homogeneous electrochemical potential than that of the control film, in accordance with a decrease in the number of grain boundaries upon treatment (Fig. 1g, h). The UPS results also show that the surface band structure has changed (Fig. 1i), since the work function decreases from 4.14 to 3.83 eV, i.e., by 0.31 eV, as determined by a linear extrapolation of the secondary electron cutoff (Fig. 1i; left). This change is consistent with the shift of the surface potential probed by KPFM. Moreover, the logarithmic extrapolation of the leading edge (Fig. 1i; right) provides a value of 1.45 eV for the valence band maximum (VBM) of the control perovskite and 1.71 eV for the treated perovskite^24^. The VBM of the treated sample surface is slightly larger than the bulk bandgap of 1.56 eV^24^, which is an indication of a possible bandgap widening at the perovskite surface due to Cs incorporation upon treatment. Such surface bandgap widening can be helpful in suppressing interface recombination, and the corresponding structural origin of these interfacial changes is further investigated below.

### Structural properties of perovskite materials

**Fig. 2:**
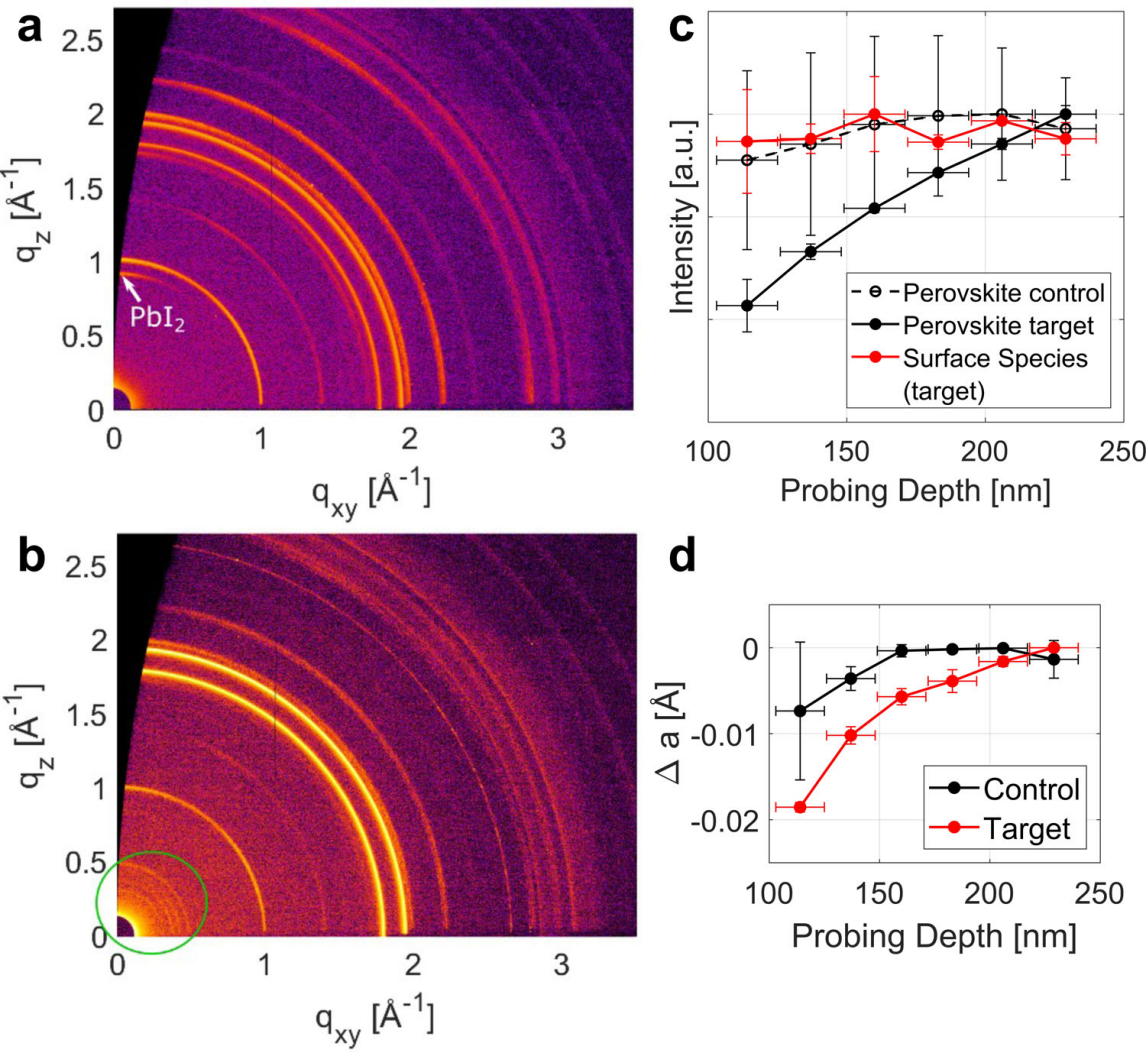
Structural characterization of perovskite films. **a**–**b** GIWAXS two-dimensional reciprocal space maps of **a** control sample, **b** treated sample at an incidence angle of 0.12°, probing the surface of the material. Low q signals of the new surface species are marked with a green circle. Measurements were done under vacuum conditions. **c** Peak intensities of different crystal phases as a function of the estimated probing depth, normalized to their respective maximum value for control and treated samples. The intensity of surface species is approximately the same for the probed depth (red). **d** Relative change in pseudo-cubic unit cell parameters as a function of probing depth. Values were calculated from radial profiles of reciprocal space maps and averaged over two sets of samples. GIWAXS probing depth was varied by changing the angle of incidence of the X-ray beam from between 0.12 and 0.3°. The error in the probing depth (horizontal error bars) was estimated from the angular error due to the surface roughness of the sample. The vertical error bars were determined from the standard deviation from repeated measurements on two sets of samples.

The structural properties of the perovskite films were analyzed by grazing incidence wide-angle X-ray scattering (GIWAXS; Fig. 2a–d and Supplementary Fig. 7a, b)^25^. Apart from the perovskite phases, unreacted PbI_2_ (*q* = 0.9 Å^−1^) and, in some cases, even hexagonal phases, can be detected on the surface of the control films (Fig. 2a, Supplementary Fig. 7a). Conversely, in the treated sample, the PbI_2_ peaks almost completely disappear while additional low-q signals appear located at *q* = 0.5 Å^−1^ and below (Fig. 2b, Supplementary Fig. 7b). These low-q signals might be ascribed to new unknown surface species, the CsI–DB21C7 complex. The new peak at *q* = 0.5 Å^−1^ also occurs in the powder X-ray diffraction pattern (pXRD; Supplementary Fig. 7c) of perovskite films after treatment. Since a broad peak around *q* = 0.5 Å^−1^ has also been observed in the pXRD pattern of CsI–DB21C7 powder, this peak might therefore arise from the formation of crystals of CsI–DB21C7. To verify this hypothesis, we generated a crystal structure of CsI–DB21C7 based on previously reported crown ether crystal structures^26^ (Supplementary Fig. 7d) and optimized it by using density functional theory (DFT) calculations (computational details provided in the Methods). The simulated XRD patterns reveal that the first peak is positioned at *q* = 0.5 Å^−1^ (Supplementary Fig. 7c) in good agreement with the experimental observations. Therefore, we propose that the new peak at *q* = 0.5 Å^−1^ (Fig. 2b) corresponds to the CsI–DB21C7 complex, while the three peaks below *q* = 0.5 Å^−1^ could arise from slightly different polymorphs, i.e., different complexes in the solid state, which are known to occur^20^.

We further analyzed the GIWAXS as a function of the incident angle to assess the structural properties at the surface as well as the bulk (Supplementary Fig. 7a, b). Lower incidence angles imply a smaller probing depth in the material and thus increased surface sensitivity (Supplementary Fig. 7e). For the target Cs-doped film, the intensities of the perovskite peaks increase with the probing depth, indicating that the modified surface has a lower perovskite content than the bulk of the material (Fig. 2c). In contrast, the control sample shows only a slight increase in the perovskite signal with increasing probing depth (Fig. 2c), which is much less pronounced than the target sample, indicating a homogeneous perovskite composition. Moreover, much more PbI_2_ is found in the control sample than in the treated sample. In the former, there is more PbI_2_ near the surface than in the bulk; however, in the latter, the PbI_2_ content increases with incident angle in a similar manner to the perovskite signal, indicating a homogeneous distribution throughout the perovskite film, and the total amount is greatly reduced upon surface modification. This is expected to enhance the stability of the Cs-doped perovskite films^11^. The peak intensity of the surface species associated with the CsI–DB21C7 complex remains approximately the same throughout the probed range of incident angles (Fig. 2c), in accordance with it being located exclusively on the surface of the film. This is not surprising considering that the complex is too large to diffuse into the perovskite lattice. The X-ray reflectivity (XRR) scans (Supplementary Fig. 7f) yield an estimated minimum thickness of the surface layer (i.e., crystallite size in the vertical direction) of approximately 15 nm.

The hexagonal 4H phase of FAPbI_3_ can also be found near the film surface of some of the control samples, but in none of the target samples (Supplementary Fig. 7a, b). This might originate from incomplete conversion to the perovskite (α) phase, since the structure of the 4H phase is a combination of δ (face-sharing) and α (corner-sharing) phases, suggesting that the 4H phase could constitute a possible intermediate during the conversion from δ to α phase. The 4H polytype, however, is converted into the α-phase perovskite structure at the Cs-rich surface of the treated samples. To understand the effect of Cs^+^ incorporation on the 4H polytype, we calculated the relative energies of the 4H polytype and the α phase as a function of Cs^+^ concentration (Supplementary Fig. 8a). The calculation reveals that Cs^+^ doping with increasing concentrations significantly decreases the stability of the 4H phase as compared to α-FAPbI_3_. Therefore, the formation of a Cs-rich surface benefits the stabilization of α-FAPbI_3_. The formation of a wider-bandgap Cs-substituted perovskite at the interface between the bulk perovskite and the hole transporting material would also be beneficial for the hole extraction and inhibition of charge recombination. Furthermore, with increasing angle of incidence, i.e., increasing penetration depth, the perovskite unit cell parameter increases (Fig. 2d). This effect is much more pronounced in the target sample, which indicates a smaller unit cell near the surface than in the bulk due to the higher concentration of Cs^+^, which is smaller than FA^+^, corroborating the gradient of incorporation of Cs^+^. The XPS (Supplementary Fig. 4c, d) and time-of-flight secondary ion mass spectrometry (TOF-SIMS; Supplementary Fig. 6c) depth profiles further verify the infusion of Cs^+^ ions (as detailed in Supplementary Note 1).

### Elucidation of the Cs doping and passivation at the atomic level

To gain atomic-level insight into the effect of Cs^+^ ion-complexed crown ether on hybrid perovskite materials, we performed solid-state nuclear magnetic resonance (ssNMR) experiments. Previously, various key phenomena in hybrid perovskite systems have been unraveled using ssNMR, such as halide mixing^27^, phase segregation^28^, cation incorporation^28,29^, as well as the effect of larger organic moieties^30–32^ on these complex organic–inorganic photovoltaic materials. In the present case, the chemical shift of ^133^Cs NMR can distinguish the incorporation of Cs^+^ ions into the perovskite^28^ from its association with the crown ether^33^, and the quadrupolar coupling of ^14^N in FA cations^34^ is extremely sensitive to the symmetry of tumbling of the organic cation and hence to distortions of the cuboctahedral cavity^30,31^.

**Fig. 3:**
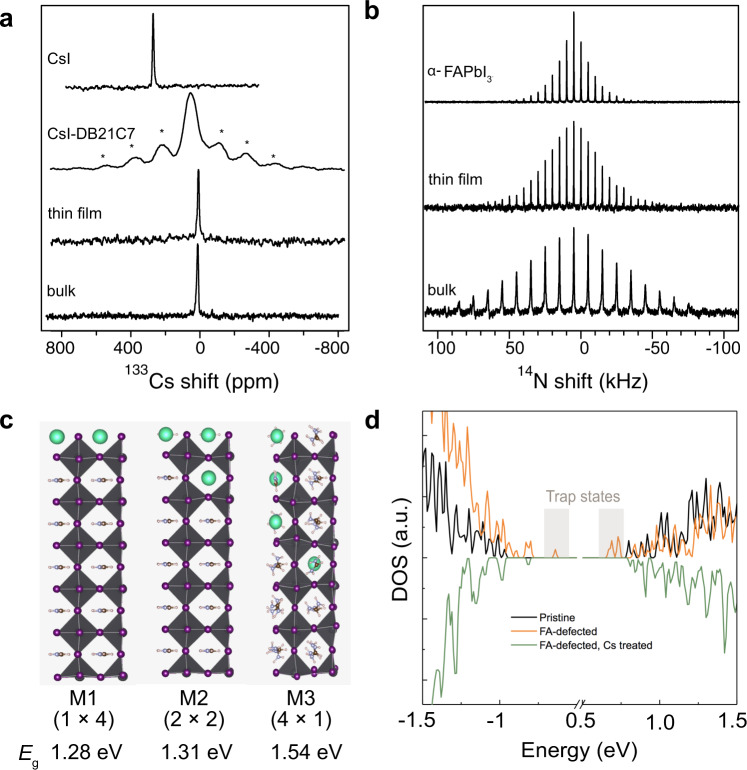
Atomic-level effects of Cs doping on the properties of FAPbI_3_. Solid-state NMR measurements at 21.1 T recorded with a Hahn-echo pulse sequence: **a** ^133^Cs spectra at 20 kHz magic angle spinning (MAS) of crystalline CsI, the CsI–DB21C7 powder, the FAPbI_3_ thin film treated with CsI–DB21C7 (thin film) and a sample of FAPbI_3_ mixed with CsI–DB21C7 complex (bulk). **b** ^14^N spectra at 5 or 10 kHz MAS and ambient temperature of the treated samples and powders prepared by mechanochemical synthesis of pure FAPbI_3_. **c** Schematic representation of inhomogeneous (1 × 4 and 2 × 2) and homogeneous (4 × 1) models (M1–M3) of Cs-doped FAPbI_3_ structures and their DFT-calculated band gap (*E*_g_) values associated with each configuration. The difference in the bandgap between the experimental and theoretical values is mainly due to finite temperature effects (more details provided in the supplementary materials). **d** Density of states of FAI-terminated pristine FAPbI_3_, FAPbI_3_ containing a FA^+^ vacancy and Cs^+^-treated FAPbI_3_ with a FA^+^ vacancy. For readability purposes, the density of states has been mirrored for the treated system.

The ^133^Cs spectrum of CsI shows a single sharp peak at 271 ppm indicating that the Cs^+^ ion is in a well-defined cubic environment, whereas upon reaction with DB21C7 a broad ^133^Cs resonance is observed with several spinning sidebands (Fig. 3a, CsI and CsI–DB21C7). This is consistent with a distribution of asymmetric local Cs^+^ environments arising from coordination by the crown ether. The ^133^Cs and ^14^N NMR spectra of mechanochemically prepared^35^ FAPbI_3_ with 10 at% CsI–DB21C7 demonstrate incorporation of Cs^+^ into the perovskite. Specifically, a sharp ^133^Cs resonance is observed at 15 ppm (Fig. 3a, bulk). Using the linear dependence of the ^133^Cs shift on the Cs^+^ concentration of *δ* = 122*x* + 0.6 ppm based on the previous data (Supplementary Fig. 9)^28^, a concentration of 12 at% is predicted from the experimental shift. There is no evidence of the signal corresponding to the Cs^+^ in the crown ether complex (CsI–DB21C7; Fig. 3a) although, due to its broadness, the intensity of this signal would be far lower. The ^14^N spectrum exhibits a spinning sideband manifold with a full width at half maximum (FWHM) of 76 kHz (Fig. 3b), compared to pure FAPbI_3_ which has a FWHM of 19 kHz at room temperature. This indicates that the cuboctahedral cavity is more distorted upon exposure of the FAPbI_3_ film to CsI–DB21C7, which is consistent with the incorporation of Cs^+^ ions into the perovskite lattice. For the thin-film sample with gradient Cs doping, ssNMR further confirms the incorporation of Cs^+^. Specifically, a sharp ^133^Cs signal can be observed at 8 ppm, which would correspond to a lower Cs^+^ concentration of 6 at% (Fig. 3b, thin film). The FWHM of the ^14^N sideband manifold is 48 kHz; this is intermediate between those of pure FAPbI_3_ and the sample mechanosynthesized with CsI–DB21C7, which again is consistent with an intermediate Cs^+^ concentration.

The concentration of Cs^+^ in the doped layer of FAPbI_3_ is further determined by the propensity for release of Cs^+^ from CsI–DB21C7 into the perovskite, which was assessed by DFT calculations. The complexation energy of CsI–DB21C7 was calculated and compared to that of FAI–DB21C7 and PbI_2_–DB21C7 (Supplementary Table 1). Complexation energies of DB21C7–Cs^+^ (−2.22 eV) and DB21C7–FA^+^ (−1.68 eV) show a higher stability of the Cs^+^ complex with respect to the FA^+^ complex, yet indicate that the formation of a crown ether–FA^+^ adduct, DB21C7–FA^+^, is also energetically favorable. In addition, the large complexation energy of DB21C7–Pb^2+^ (−8.93 eV) strongly suggests that such complexes could form on the surface of FAPbI_3_, which would be further beneficial for the suppression of uncoordinated Pb defects.

The dissociation of Cs^+^ ions from the crown ether and its incorporation into the perovskite phase are expected to cause changes in the electronic structure of FAPbI_3_, which are further analyzed via DFT calculations to assess the benefits of the gradient doping. Accordingly, we considered 2 × 2 × 6 supercells of 16.7% Cs-doped FAPbI_3_ with three distinct distributions of Cs^+^ (models M1–M3) (Fig. 3c, Supplementary Fig. 8b–e), which closely corresponds to the experimental concentrations while enabling a comparison of different distributions and pure FAPbI_3_. Although the compositions are slightly different and we recognize the limitation of the periodic models, the effect of the different doping distributions can nevertheless be inferred. The M1 (1 × 4) model was constructed by replacing an entire 2 × 2 layer of FA^+^ by Cs^+^. Similarly, the M2 (2 × 2) model was constructed by replacing 2 FA^+^ cations in each of two adjacent layers by Cs^+^ ions. Lastly, the M3 (4 × 1) model was constructed by replacing one FA^+^ cation by Cs^+^ in each of the 4 layers. Models M1 (1 × 4) and M2 (2 × 2) represent inhomogeneous surface treatment of FAPbI_3_, which do not show a significant difference in the bandgap (1.28 eV and 1.31 eV, respectively, as shown in Fig. 3c and Supplementary Fig. 8e) compared to the pure FAPbI_3_ (1.32 eV), in accordance with the experimental findings. The compositions represented by models M1–M2 feature an FA-rich and a Cs-rich domain. The band edges are found to be dominated by the Pb and I contributions from the FA-rich region, which remains undistorted, hence maintaining the band gap of FAPbI_3_. However, the bandgap of the Cs-rich region is, as demonstrated by the corresponding projected density of states (Supplementary Fig. 8c, d), larger than that of the FA-rich region, allowing for a more efficient charge extraction and lower recombination. On the contrary, in the case of the more homogeneously Cs-doped model M3 (4 × 1), a significant increase in the bandgap is observed (Fig. 3d) due to considerable structural distortion (octahedral tilting away from 90° to more orthorhombic environments), which leads to shifts of both the VBM and conduction band minimum (CBM; Supplementary Fig. 8e).

To investigate potential benefits of Cs^+^ incorporation for defect passivation at the atomic level, we performed a DFT study of a FA^+^ vacancy-containing surface of FAPbI_3_. When the FA^+^ vacancy is present at the perovskite surface (Fig. 3d, Supplementary Fig. 8f, g), localized trap states were introduced in the vicinity of both the VBM and the CBM, as a result of strong structural distortions of the surface induced by the vacancy. Upon the vacancy passivation by Cs^+^, the VBM and the CBM states delocalized and the gap became larger, resulting in a direct passivation effect of the surface defects by Cs^+^. In a similar fashion, incorporation of Cs^+^ ions in the bulk of the perovskite can passivate defects, thus hindering the recombination and improving the photovoltaic performances. However, the gradient structure enables this without compromising the resulting optoelectronic properties, which is more beneficial for photovoltaic applications.

### Photovoltaic performances and device physics

**Fig. 4:**
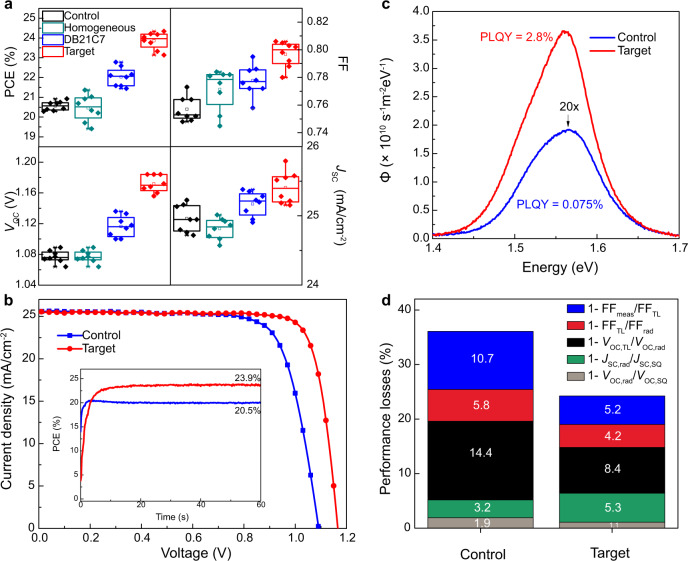
Photovoltaic device performance. **a** Photovoltaic metrics of devices without Cs doping (control), with homogeneous Cs doping, after treatment with DB21C7–CsI (target) and after just crown ether (DB21C7) treatment. **b** *J–V* curves of the champion control and target PSCs. The inset shows the maximum power point tracking data. An anti-reflection coating was used for the champion devices. **c** Photoluminescence spectral photon flux Φ measured on full devices. **d** Breakdown of losses derived from the *J–V* curves in **b**; radiative *J*_SC_ and *V*_OC_ losses are shown in green and grey, respectively; non-radiative *V*_OC_ and FF losses in black and red; transport losses affecting FF in blue.

The device performance of the corresponding perovskite solar cells was investigated using the conventional configuration of FTO/compact TiO_2_ (~60 nm)/ mesoporous Li-TiO_2_:perovskite composite layer (~150 nm)/perovskite upper layer (~650 nm)/ spiro-OMeTAD (~150 nm)/Au (~70 nm)^36^. The perovskite layers were based on both FAPbI_3_ and a more widely used FAPbI_3_-rich composition of (FAPbI_3_)_0.97_(MAPbBr_3_)_0.03_, to illustrate the generality of the approach. The DB21C7–CsI target treatment is compared with undoped control samples, homogeneously Cs^+^ doped samples and DB21C7 treated samples. Experimental details are provided in the Methods and photovoltaic (PV) performance is illustrated in Fig. 4 and Supplementary Figs. 10–12. The treatment significantly improves the performance of PSCs compared to that of the controls (without doping; Fig. 4a). This is particularly reflected in the *V*_OC_, which is improved from 1.08 ± 0.01 V to 1.17 ± 0.01 V and the fill factor (FF), which improves from 75.7 ± 0.9% to 79.7 ± 0.9%, resulting in a significant improvement of the power conversion efficiency (PCE) (average) from 20.56 ± 0.21% to 23.62 ± 0.43%. Current density–voltage (*J–V*) curves of the champion devices (Fig. 4b and Supplementary Fig. 10) show that the target (gradient-Cs-doped) device exhibited a *V*_OC_ of 1.17 V, a short-circuit current (*J*_SC_) of 25.50 mA cm^−2^, a fill factor of 81.9%, and a PCE of 24.30% for (FAPbI_3_)_0.97_(MAPbBr_3_)_0.03_ composition, while the control device showed an overall PCE of 21.20% with a *V*_OC_ of 1.09 V, a *J*_SC_ of 25.60 mA cm^−2^, and a FF of 75.9%. We further ascertained these values by recording scan-speed-independent maximum power point tracking (MPP) measurements (Fig. 4b, insert) corresponding to PCEs of 20.5% and 23.9% for the control and target PSCs, respectively.

The target PSCs show much higher performance than for the homogeneous Cs-doped perovskites^17,18^, which further demonstrates the advantages of the approach (Fig. 4a). Furthermore, after releasing the Cs^+^ ions, the remaining crown ether assemblies also play a passivating role as a modulator of surface defects, further improving the PSC performance (Fig. 4a). This is in accordance with the binding mode previously assessed by solid-state NMR spectroscopy and DFT calculations. Consequently, the synergistic effect of the Cs^+^ gradient structure and crown ether surface modulation contribute to the significant improvement of photovoltaic performances through a multimodal host–guest complexation approach, i.e., beneficially affecting both bulk and surface properties. This strategy could also be employed in the delivery of other alkali metal cations^19,20^, which we demonstrate by using Rb^+^ ions (Supplementary Fig. 11); however, we note that since, unlike Cs^+^, Rb^+^ has been shown not to incorporate into bulk perovskites by occupying A cation sites, rather passivation of grain boundary defects by Rb^+^-rich phases is the most likely mechanism in this case, as previously indicated^28^. Moreover, we further illustrate the generality of the approach by fabricating different perovskite compositions, both Br/MA-based and Br/MA-free FAPbI_3_ (Supplementary Fig. 12a). We note that the perovskite compositions based on excess A-cations (such as FAI) are not the subject of this investigation. The *J*_SC_ value obtained from the *J–V* characteristics matches (within 2%) the integrated currents obtained from the external quantum efficiency (EQE; Supplementary Fig. 12b), excluding any significant spectral mismatch between our simulator and the AM1.5 G solar source.

We further investigated the origin of the improved photovoltaic performance by performing time-resolved photoluminescence (TRPL) measurements to study the carrier transport and recombination at the perovskite layer deposited on microscope glass substrates. To evaluate the TRPL data we applied a kinetic model described in our previous study^37^. Assuming a negligible surface recombination rate, we calculate the average monomolecular bulk recombination constant *k*_1_ to be 2.3 × 10^5^ s^−1^ for the control and 1.4 × 10^5^ s^−1^ for the target Cs-doped film (the TRPL data and the fit curves are shown in Supplementary Fig. 13). This indicates that the nonradiative recombination channels in perovskite films have been suppressed by the treatment, which was further demonstrated by the increased perovskite emission in the cathodoluminescence (CL) mapping (Supplementary Fig. 14) and decreased ideality factor *n* (from 1.51 to 1.41; Supplementary Fig. 15). These changes are expected to be further reflected by the quasi-Fermi level splitting (∆*E*_F_) which represents the upper limit for the *V*_OC_^38,39^. For this we measured the absolute spectral photon flux Φ in an integrating sphere (Fig. 4c) and derived the PL quantum yield (PLQY) according to the method described in de Mello et al.^40^, ∆*E*_F_ was determined by Δ*E*_F_ = *q* *V*_oc,rad_ + *k*_B_*T* ln(PLQY), where *q* is the elementary charge, *V*_oc,rad_ the radiative limit of *V*_OC_, *k*_B_ the Boltzmann constant, and *T* the device temperature (25 °C). The determination of *V*_oc,rad_ from the absorbance spectrum (Supplementary Fig. 16) is described in detail in our previous work^37^ (the values are shown in Supplementary Table 2). Δ*E*_F_ for the control device is 1.09 eV and for the target device 1.17 eV which is in very good agreement with the measured *V*_OC_ (Fig. 4a). This confirms that the *V*_OC_ improvement originates mainly from a ∆*E*_F_ increase, and thus from a reduction in non-radiative recombination. Further analysis of the PL spectra^41^ reveal no significant contribution arising from Urbach energy and radiative-recombination *V*_OC_ limit differences (Supplementary Fig. 16 and Supplementary Table 2).

The origin of the performance improvement is further analyzed by investigating the devices’ diode characteristics (as detailed in Supplementary Note 2, Supplementary Figs. 17 and 18, and Supplementary Tables 3 and 4). The analysis of performance losses (Fig. 4d) shows that a significant improvement in non-radiative losses is observed, decreasing from 20.2% in the control device to 12.6% in the target device, confirming the role of the reduction of non-radiative recombination as the main driver for the performance improvement observed here. Meanwhile, a notable improvement in transport losses (10.7% to 5.2%) is also observed, arising from decreases in series resistance and ideality factor, which are both traced to the suppression of interfacial defects or barriers^42^. The suppression of interfacial defects is also relevant to the overall stability of the resulting devices.

### Stability and environmental impact

**Fig. 5:**
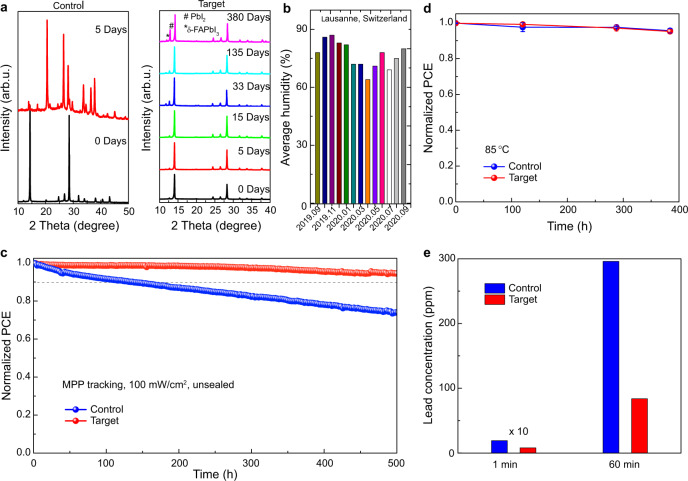
Stability and enviromental impact. **a** Ambient stability of the control perovskite film (left) and target perovskite film (right) based on the evolution of the XRD patterns of the perovskite films stored in an ambient environment for various times. **b** The average relative humidity during the aging process in Lausanne, Switzerland. **c** Maximum power point tracking measured with the unencapsulated device under full solar illumination (AM 1.5 G, 100 mW cm^−^^2^ in N_2_, 25 °C). **d** Thermal stability of perovskite devices at an elevated temperature of 85 °C. **e** Assessment of lead leakage upon immersing solar cells into water. The PCE refers to the steady-state efficiency. The concentration of lead in water was determined by inductively coupled plasma optical emission spectroscopy (ICP-OES) measurement.

We investigated the stability (shelf life) of the perovskite films and the corresponding devices by exposing the films to an ambient air environment of 60 ± 10% relative humidity and temperature of 25 ± 1 °C, respectively (Fig. 5a–b). The monthly average humidity during the aging test is presented in Fig. 5b. The Cs-complex surface treatment was found to significantly enhance the stability under these conditions, as the target perovskite film was stable in air for more than one year (380 days), while the control film degraded completely within 5 days. In addition, we probed the long-term operational stability of the unencapsulated PSCs under one-sun illumination by maximum power point tracking (Fig. 5c). The target device exhibited very high photostability, maintaining >95% of its initial PCE over 500 h of illumination, whereas the control device degraded to 80% of initial PCE in the first 300 h. The enhanced ambient and operational stability can be attributed to a lower concentration of defects at the interface between the hole transport material and the perovskite absorber, as well as to fewer impurities (including excess PbI_2_^11^ and non-perovskite polytypes) on the surface and in the bulk of FAPbI_3_, caused by Cs^+^ infusion via the host–guest complexation and the corresponding nanostructures. Finally, the devices maintain >90% of their operational stability over 300 h at elevated temperatures of 85 °C (Fig. 5d). Under these accelerated aging conditions, the stabilities of the control and treated devices are comparable, which is likely due to the bulk becoming more homogeneous, whereas the surface still retains a local Cs-rich gradient structure as indicated by the elemental mapping (Supplementary Fig. 19). Moreover, the capacity for competitive complexation of Pb cations (Supplementary Table 1) enables lead leakage to be suppressed, which has been probed by immersing the solar cells in water and quantifying the lead content, showing that presence of the crown ether hosts mitigates the detrimental environmental impact of lead (Fig. 5e). This has been shown for the optimal concentrations of the crown ether used in the solar cell fabrication, which is rather low, and increasing this content is likely to further suppress the environmental impact of lead. Treatment with Cs-crown ether complexes therefore provides a versatile approach for the enhancement of performance and stability of perovskite-based devices, while reducing their environmental impact by virtue of multimodal host–guest complexation, suggesting wide-ranging applications.

## Methods

### Materials

Lead iodide (PbI_2_) is purchased from Alfa Aesar. Formamidinium iodide (FAI) and titanium dioxide paste (TiO_2_−30 NRD) are purchased from Greatcell. 2,2′,7,7′-Tetrakis[*N*,*N*-di(4-methoxyphenyl)amino]−9,9′-spirobifluorene (Spiro-OMeTAD) is purchased from Xi’an Polymer Light Technology Corp. Methylammonium lead tribromide (MAPbBr_3_) purchased from Share Chem. Ultra-dry dimethylformamide (DMF), dimethyl sulfoxide (DMSO), ethanol (EtOH), and chlorobenzene (CB) are purchased from Acros. Dibenzo-21-crown-7 (DB21C7), cesium iodide (CsI), cesium bromide (CsBr), cesium fluoride (CsF), cesium chloride (CsCl), 4-*tert*-butyl pyridine (TBP), lithium bis(trifluorosulfonyl)imide (LiTFSI), acetonitrile (ACN), acetyl acetone, titanium diisopropoxide bis(acetylacetonate) 75 wt.% in isopropanol, and methylammonium chloride (MACl) are purchased from Sigma-Aldrich. Fluorine-doped tin oxide (FTO) (10 Ω/sq) conductive glass is purchased from Nippon Sheet Glass (NSG). All the chemicals are used as received without further purification.

### Synthesis of CsX–DB21C7 complex

CsX–DB21C7 complex (X = F, Cl, Br, I) is synthesized by mixing DB21C7 and CsX with 1:1.2 mole ratio in 1 mL dry chlorobenzene and stirring at 50 °C for 7 days. The solution was filtered to obtain a nearly quantitative amount of the complex, which was used for device fabrication. The concentration of CsX–DB21C7 solution is controlled by the amount of crown ether.

### Preparation of perovskite precursor solution

For (FAPbI_3_)_0.97_(MAPbBr_3_)_0.03_ precursor solution preparation, a mixture of PbI_2_ (735 mg), FAI (252 mg), MAPbBr_3_ (21 mg), and MACl (30 mg) is dissolved in 1 mL mixed solution of DMF and DMSO (volume ration of DMF/DMSO of 4:1). For the homogeneously doped perovskite, 50 μL CsI solution (390 mg/mL in DMSO) is added to the 1.1 mL above (FAPbI_3_)_0.97_(MAPbBr_3_)_0.03_ precursor solution to prepare ~4 at% Cs-doped perovskite film. For pure FAPbI_3_ precursor solution preparation, a mixture of PbI_2_ (755 mg), FAI (252 mg), and MACl (40 mg) is dissolved in 1 mL mixed solution of DMF and DMSO (volume ration of DMF/DMSO of 4:1).

### Device fabrication

FTO substrates are cleaned using 2% Hellmanex aqueous solution, deionized water, acetone, and ethanol consecutively by sonicating for 20 min for each solvent. After drying with compressed air, UV-Ozone treatment for 15 min is applied for further cleaning. Compact TiO_2_ (c-TiO_2_) is deposited on top of FTO using spray pyrolysis method: the substrates are preheated to 450 °C; a precursor solution of titanium diisopropoxide bis(acetylacetonate), 75 wt.% in isopropanol is diluted with ethanol with a volume ratio of 1:9 and the addition of 4% volume ratio of acetylacetone. After spray pyrolysis, the FTO/c-TiO_2_ substrate is allowed to heat at 450 °C for 30 min before cooling down to room temperature. Mesoscopic TiO_2_ (mp-TiO_2_) is applied by spin-coating a diluted solution of 30 NR-D paste (mass ratio of paste:ethanol = 1:10) at 4000 rpm with the acceleration of 2000 rpm/s, followed by sintering at 450 °C for 60 min to obtain mp-TiO_2_ substrate. 0.1 M LiTFSI solution is then coated on the mp-TiO_2_ according to our previous report^36^. Another sintering process at 450 °C for 30 min is performed. The Li-treated mp-TiO_2_ is transferred to dry box for device fabrication intermediately. The perovskite active layer is deposited using antisolvent method. The corresponding perovskite precursor solution is deposited on the freshly prepared FTO/c-TiO_2_/mp-TiO_2_ substrate with a two-step spin-coating method at 1000 rpm for 10 s and followed by 5000 rpm for 25 s. 200 µL of diethyl ether is applied at the last 10 s. After spin-coating, the substrate is allowed to anneal at 150 °C for 10 min, then 100 °C for 10 min. The whole procedure is done in a glovebox filled with dry air. The CsX–DB21C7 treatment is conducted by coating the as-prepared perovskite with a solution of CsX–DB21C7 complex solution (100 µL) with various concentration. The solutions are kept on surface of the perovskite film for 2 s, and substantially spin-coated at 4000 rpm for 30 s. The treated perovskite films are annealed at 100 °C for 5 min. The optimized concentration is 8 mg/mL. Later, the perovskite films are washed with 100 μL CB five times (the effects of the antisolvent are excluded by comparison in a control experiment; Supplementary Fig. 20). The doped spiro-OMeTAD solution was spin-casted on the surface of the perovskite at 4000 rpm with acceleration of 2000 rpm/s for 30 s. Spiro-OMeTAD is dissolved in chlorobenzene with a concentration of 90 mg/ml, which is doped by 23 μl LiTFSI (520 mg/mL in CH_3_CN) and 39.5 μl 4-*tert*-butyl pyridine, and 10 μl FK209 (375 mg/mL in ACN). The whole procedure is carried out in a glovebox filled with dry air (temperature < 28°C; relative humidity <15%). The device fabrication is completed with deposition of gold electrode (~70 nm) by evaporation.

### Photovoltaic performance measurements

The prepared perovskite solar cells were measured using a 300 W Xenon light source from Oriel. The spectral mismatch between AM 1.5 G and the solar simulator was calibrated by a Schott K113 Tempax filter (Prazosopms G; as & Optik GmbH). Before each measurement, the exact light intensity was determined using a calibrated Si reference diode (certified and calibrated by Newport Corporation PV Lab, Bozeman, MT, USA) equipped with an infrared cut-off filter (KG-3, Schott). Keithley 2400 is used for the current–voltage scan by applying an external voltage bias and measuring the response current with a scan rate of 50 mV/s. The device area was 0.25 cm^2^ (0.5 cm × 0.5 cm). The cells were masked with a black metal mask with an area of 0.16 cm^2^. No preconditioning (e.g., bias and light soaking) was used for the photovoltaic measurement. External quantum efficiency (EQE) was recorded with a commercial apparatus (Aekeo-Ariadne, Cicci Research s.r.l.) based on a 300 W Xenon lamp. Stability of the cells was measured under a white light-emitting diode lamp with biologic MPG2 potentiostat and was performed under open air. The device area is masked to around 0.13 cm^2^. The spectral mismatch between AM 1.5 G and the solar simulator was calibrated by a Schott K113 Tempax filter, whose light intensity is calibrated with a silicon diode. The light intensity is around 100 mW cm^−2^, and the actual current is adjusted according to in-time calibration result from the silicon diode. The stability data is acquired from MPP tracking of unencapsulated device under a continuous nitrogen flow at 25 °C.

### GIWAXS and pXRD measurements

X-ray scattering experiments were done at beamline P08 at PETRA III (DESY) with a photon energy of 18 keV under nitrogen atmosphere. The beam size was 100 µm in vertical direction and 500 µm in horizontal direction. GIWAXS data were measured with a PerkinElmer XRD1621 area detector under various angles of incidence. Powder X-ray diffraction (pXRD) spectra were recorded on an X’Pert MPD PRO (PANanalytical) equipped with a ceramic tube providing Ni-filtered (Cu anode, *λ* = 1.54060 Å) radiation and a RTMS X’Celerator (PANalytical).

### SEM and EDX measurements

The morphologies and element mapping of the films were characterized using high-resolution scanning electron microscope (Zeiss Merlin) with an in-lens detector.

### STEM-EDX measurements

Transmission electron microscopy (TEM) investigations were undertaken to study the microstructure and chemical composition using a Thermo Fisher Tecnai Osiris electron microscope under 200 kV accelerating voltage. Energy dispersive X-ray spectroscopy (EDX) elemental mapping were carried out using the TEM-attached 4 super-detectors, combining with high angle annular dark field (HAADF) images in scanning transmission electron microscopy (STEM) mode with a spot size of 0.5 nm and step size of 1.0 nm. TEM lamellae are extracted and thinned down using Focused Ion Beam (Gemini NVision 40) at 30 kV and finally polished at 5 kV.

### PL, TRPL, and UV–Vis measurements

UV–Vis absorptions were measured using Varian Cary 500 spectrometer (Varian USA). Photoluminescence lifetime (TCSPC) was measured using an Edinburgh Instruments lifespec II fluorescence spectrometer; a picosecond pulse diode laser (EPL-510, excitation wavelength 510 nm, pulse width <60 ps, fluence <3 nJ/cm^2^) was used. Photoluminescence spectral photon flux was measured using an Andor Kymera 193i spectrograph and a 660 nm continuous-wave laser set at 1-Sun equivalent photon flux (1.1 µm beam full-width half-maximum, 632 µW); photoluminescence was collected at normal incidence using a 0.1 NA, 110 µm-diameter optical fiber.

### XPS measurements

XPS measurements were performed with a PHI VersaProbe II scanning XPS microprobe using a monochromatic Al Ka *X*-ray of 24.8-W power with a beam size of 100 mm. Core-level signals were obtained at 45° take-off angle. All peaks were calibrated using C 1 *s* peak at 284.8 eV to correct charge shift of binding energies. Curve fitting was performed using the PHI MultiPak software. Depth profiling etching speed was calibrated using Si as standard. The Cs diffusion length is estimated by combing the I 3*d* level XPS depth profiles and cross-sectional SEM image of treated perovskite film. In detail, the average thickness of treated perovskite film is calculated to be 820 ± 80 nm from the cross-sectional SEM in Supplementary Fig. 4b. The I *3d* signal can only be detected at layer 22 in the XPS depth profiles. So, the sputtering thickness for each layer is 37.3 ± 3.7 nm. Considering the Cs *3d* signal can only be detected at layer 13, the Cs diffusion length in the treated perovskite is calculated to be 484 ± 49 nm. An ultraviolet photoelectron spectrometer (UPS) equipped with a He-I source (*hν* = 21.22 eV) (AXIS Nova, Kratos Analytical Ltd, UK) was used to determine the valence band energy and Fermi-level.

### Solid-state NMR measurements

Room temperature ^133^Cs (118.04 MHz) and ^14^N (65.37 MHz) NMR spectra were recorded on a Bruker Avance 400 MHz Neo 21.1 T spectrometer equipped with a 3.2 mm low-temperature CPMAS probe. ^133^Cs shifts were referenced to 1 M aqueous solution of CsCl, using solid CsI (*δ* = 271.05 ppm) as a secondary reference. ^133^Cs spectra were recorded with a Hahn echo and the following recycle delays: CsI, 5 s; CsI-crown ether complex, 1 s; mechanosynthesised and thin film perovskite samples, 15 s. ^14^N spectra were acquired with a Hahn echo and a repetition time of 53 ms. ^14^N spectra were referenced to solid NH_4_Cl (0 ppm) at 298 K.

### Liquid ^1^H NMR measurements

^1^H NMR spectra of Cs–DB21C7 complex was conducted on a proton nuclear magnetic resonance spectrometer (NMR, Avance 400, Bruker) using dichloromethane-*d*_*2*_ as solvent at ambient temperature (300 K).

### TOF-SIMS measurements

The TOF-SIMS measurements (Model TOF-SIMS V, ION-TOF GmbH) were performed with the pulsed primary ions from a C_60_ (10 keV) for the sputtering and a Bi^+^ pulsed primary ion beam for the analysis (25 keV).

### Cathodoluminescence (CL) measurements

CL spectra were acquired on the Attolight ROSA 4634. CL SEM operating at 2 keV with the sample held at stage temperature of 10 K in a vacuum of <10^−7^ Torr with probe current of a few 100 s pA. CL signal integrated for 1 ms per pixel. The spectrometer centered at 900 nm with spectral range from 622 to 1176 nm. Sample is kept slightly out of focus to even out the dosis distribution in each pixel. Because the sample is very sensitive to beam damage, we ensure to expose each area only once with the electron beam, during acquisition of the CL map. SE images to retrieve morphology are acquired posteriori. Hyperspectral maps were acquired with 128 × 128 pixel resolution, a pixel size of 104 nm, and a pixel dwell (spectrum exposure) time of 1 ms. The average center emission wavelength for each sample type was determined and false colored CL emission maps were reconstructed from deconvoluted CL intensity counts in the pixel spectra by subtracting the background and fitting the center CL emission peak with a Gaussian function.

### Lead leakage test

A piece of a perovskite solar cell (substrate size: 14 × 17 mm) was immersed into 40 ml deionized water. The concentration of the Pb^2+^ were determined using a ICP-OES 5110 (Agilent) instrument.

### Computational methods

For calculating the relative energies between polytypes, we used supercells of 144 atoms for each poly-type of FAPbI_3_. To understand the effect of Cs on stabilization of cubic phase, we computed the relative energies between the cubic phase and 4H polytype by doping different Cs concentrations into cubic phase and 4H. All the structures are reported in the Supplementary Figs. 21–23. For calculating energies, we performed variable-cell DFT calculations with Perdew–Burke–Ernzerhof (PBE)^43^ functional with D3-vdW^44^ dispersion corrections. The Quantum Espresso^45^ package is used for the DFT calculations with ultra-soft pseudo-potentials for valence–core electron interactions with k-point sampling (3 × 2 × 1 grid for delta phase, 3 × 1 × 2 grid for 4H, 3 × 2 × 1 grid for 6H and 1 × 1 × 2 grid for cubic phase) with a plane wave basis set of 60 Ry kinetic energy cutoff and 420 Ry density cutoff. To obtain DFT-optimized structure of CsI–DB21C7, we have used PBEsol^46^ functional with D2-vdW dispersion corrections^44^ using the ultra-soft pseudopotentials for valence–core electron interactions and a k-point sampling (8 × 3 × 4) with a plane wave basis set of 40 Ry kinetic energy cutoff and 280 Ry density cutoff. Complexation energies of DB21C7–Cs^+^, DB21C7–FA^+^, and DB21C7–Pb^2+^ were calculated for the gas phases of the reactants and the products, based on Generalized Gradient Approximation (GGA) of density functional theory, employing the PBE functional^44^ within the CPMD package^47^. Valence–core electron interactions were modeled via norm-conserving pseudopotentials in a simulation box of 50 × 50 × 50 Å^3^ together with a 100 Ry kinetic energy cutoff. The structures of Cs-doped cubic FAPbI_3_ were optimized at DFT level using the PBEsol functional^46^ within the Quantum Espresso suite^45^. To study the effect of different doping models, 2 × 2 × 6 supercells of cubic FAPbI_3_ were used. The model 1 × 4 was constructed by replacing an entire 2 × 2 layer of FA^+^ by Cs^+^. Similarly, the model 2 × 2 was constructed by replacing 2 FA^+^ cations in 2 layers by Cs^+^ ions. Lastly, the model 4 × 1 was constructed by replacing one FA^+^ cation by Cs^+^ in each of the 4 layers. To study the effect of Cs^+^ on defects at the perovskite surface, FAI-terminated slabs of cubic FAPbI_3_ were employed. FA^+^ vacancy-containing slab was created by removing one FA^+^ from the surface. Cs^+^-treated slab with a FA^+^ vacancy was created by filling the FA^+^ vacant site by a Cs^+^ ion. A vacuum gap of 25 Å was used for all slab structures to prevent interaction of the slab images. A k-point grid of 2 × 2 × 1 (4 × 4 × 1 for post processing analyses) and ultra-soft pseudopotentials for valence core electron interactions with a plane wave basis set of 60 and 420 Ry kinetic energy cutoff for the expansion of the wavefunction and the density, respectively, were employed for both bulk and slab structures. The hybrid PBE0 functional^48^ was utilized to calculate band gaps taking also spin orbit coupling (SOC) effects into account, with norm-conserving pseudopotentials of 80 Ry wavefunction cutoff and 320 Ry density cutoff. To compare valence band maximum and conduction band minimum of different configurations, energy levels were aligned with respect to the energy levels of 5*d* orbitals of Pb atoms.

### Reporting summary

Further information on research design is available in the Nature Research Reporting Summary linked to this article.

## Supplementary information

Supplementary Information

Solar Cells Reporting Summary

## Data Availability

Data that support the findings of this study are available in Supplementary Data Files in the Supplementary Information section. All relevant data are available at 10.5281/zenodo.4768098 under the license CC-BY-4.0 (Creative Commons Attribution 4.0 International). Source data are provided with this paper.

## Source data

Source Data
