# Supplementary material for: Multimodal host–guest complexation for efficient and stable perovskite photovoltaics
Source: Nat Commun. 2021 Jun 7;12:3383. doi: 10.1038/s41467-021-23566-2 (PMC8185086; doi:10.1038/s41467-021-23566-2)
Supplement: Supplementary file 1 — Supplementary Information [file 41467_2021_23566_MOESM1_ESM.pdf]

Supplementary Materials

Multimodal host-guest complexation for efficient and stable perovskite photovoltaics

Hong Zhang[1], Felix Thomas Eickemeyer[1], Zhiwen Zhou[1], Marko Mladenović[2], Farzaneh Jahanbakhshi[2], Lena Merten[3], Alexander Hinderhofer[3], Michael A. Hope[4], Olivier Ouellette[1], Aditya Mishra[4], Paramvir Ahlawat[2], Dan Ren[1], Tzu-Sen Su[1], Anurag Krishna[5], Zaiwei Wang[5], Zhaowen Dong[6], Jinming Guo[7], Shaik M. Zakeeruddin[1], Frank Schreiber[3], Anders Hagfeldt[5], Lyndon Emsley[4], Ursula Rothlisberger[2], Jovana V. Milić[1,8*], Michael Grätzel [1*]

[1]Laboratory of Photonics and Interfaces, Institute of Chemical Sciences and Engineering, École Polytechnique Fédérale de Lausanne, Lausanne 1015, Switzerland. [2]Laboratory of Computational Chemistry and Biochemistry, Institute of Chemical Sciences and Engineering, École Polytechnique Fédérale de Lausanne, Lausanne 1015, Switzerland. [3]Institut für Angewandte Physik, Universität Tübingen, Tübingen 72076, Germany. [4]Laboratory of Magnetic Resonance, Institute of Chemical Sciences and Engineering, École Polytechnique Fédérale de Lausanne, Lausanne 1015, Switzerland. [5]Laboratory of Photomolecular Science, Institute of Chemical Sciences and Engineering, École Polytechnique Fédérale de Lausanne, Lausanne 1015, Switzerland. [6]Laboratory of Supramolecular Chemistry, Institute of Chemical Sciences and Engineering, École Polytechnique Fédérale de Lausanne, Lausanne 1015, Switzerland. [7]Laboratory for Biological Geochemistry, École Polytechnique Fédérale de Lausanne, 1015, Lausanne, Switzerland. [8]Adolphe Merkle Institute, University of Fribourg, Fribourg 1700, Switzerland. *Correspondence to: jovana.milic@unifr.ch (J.M.); michael.graetzel@epfl.ch (M.G.)

All data presented here can be accessed at DOI:10.5281/zenodo.4768098 and it is available under the license CC-BY-4.0 (Creative Commons Attribution 4.0 International).

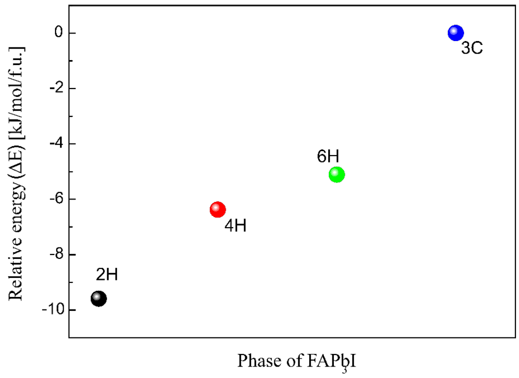

Fig. S1. DFT calculations of relative energies of different polytypes of $FAPbI_3$. 2H (δ, black), 4H (red), 6H (green), 3C (α, blue) polytype.

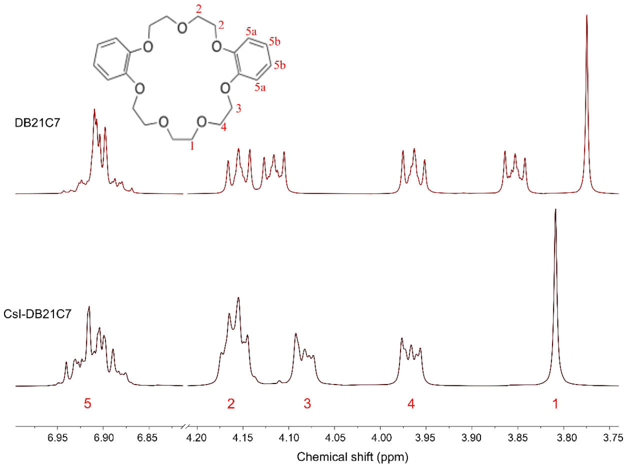

Fig. S2. ^{1}H NMR spectra of host-guest complex. ^{1}H NMR spectra of dibenzo-21-crown-7 (DB21C7) and CsI-DB21C7 complex in CD_2Cl_2. The molecular structure of DB21C7 is presented and its different protons are labeled. The assignment is based on Veldhuis et al.[1], however in this case protons 5a and 5b cannot be distinguished and the combined signal is therefore labelled as 5.

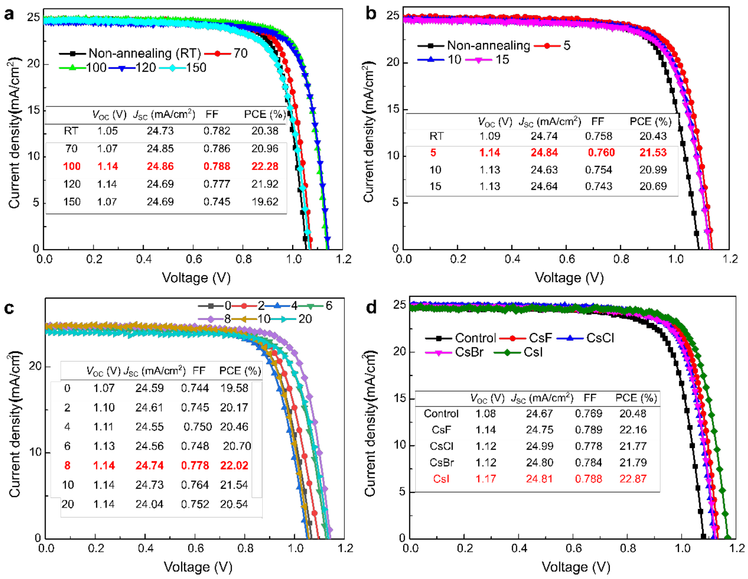

Fig. S3. Optimization of host-guest complex treatment on device performance. Photovoltaic performance of PSCs treated by 8 mg/mL CsI-DB21C7 complex solution annealed at different temperature (unit: °C) for 10 min (**a**); PSCs treated by 8 mg/mL CsI-DB21C7 complex solution annealed at 100 °C for various duration time (unit: min) (**b**); and PSCs treated by different concentration of CsI-DB21C7 complex solution (unit: mg/mL) (**c**). All the perovskite films annealed at 100 °C for 5 min. **d**, Performance of PSCs based on different Cs salts (8 mg/mL; 100 °C; 5 min). Therefore, PSCs based on 8 mg/mL CsI-DB21C7 treated perovskite with annealing at 100 °C for 5 min shows the best performance.

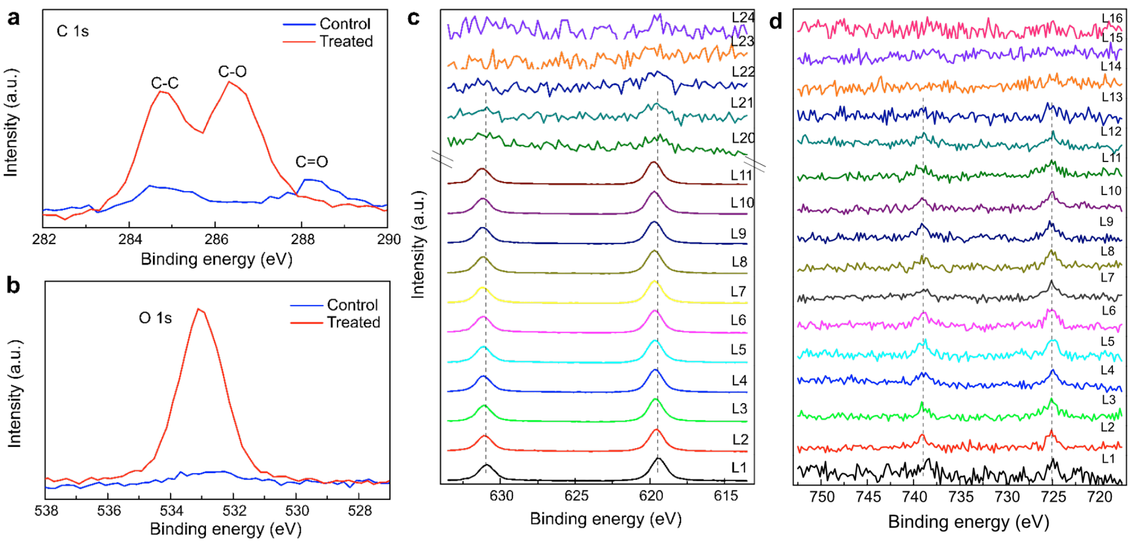

Fig. S4. XPS core-level and UPS spectra of perovskite films without and with CsI-DB21C7 treatment. XPS core-level spectra of perovskite film for C *1s* (**a**) and O *1s* (**b**). **c**, XPS depth profiles of I *3d* spectra of the treated perovskite film. **d**, Depth profiles of Cs 3*d* level XPS spectra with an approximate depth of each sputtering layer of ~40 nm.

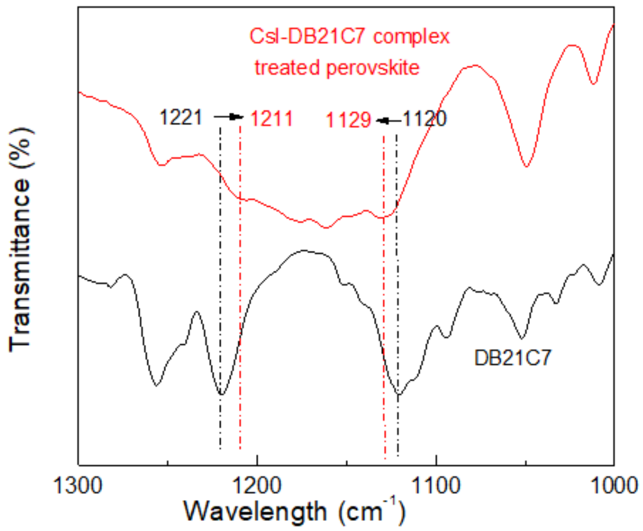

Fig. S5. Attenuated total reflection Fourier transform infrared (ATR-FTIR) spectra of CsI-DB21C7 complex-treated perovskite film, and crown ether (DB21C7) powder. We note that the peaks could not be identified precisely for the complex-treated sample (red) due to smaller concentration and the corresponding minor shifts are likely due to Pb—O coordination and hydrogen bonding, in accordance with the NMR, XPS and DFT analysis detailed in the main manuscript.

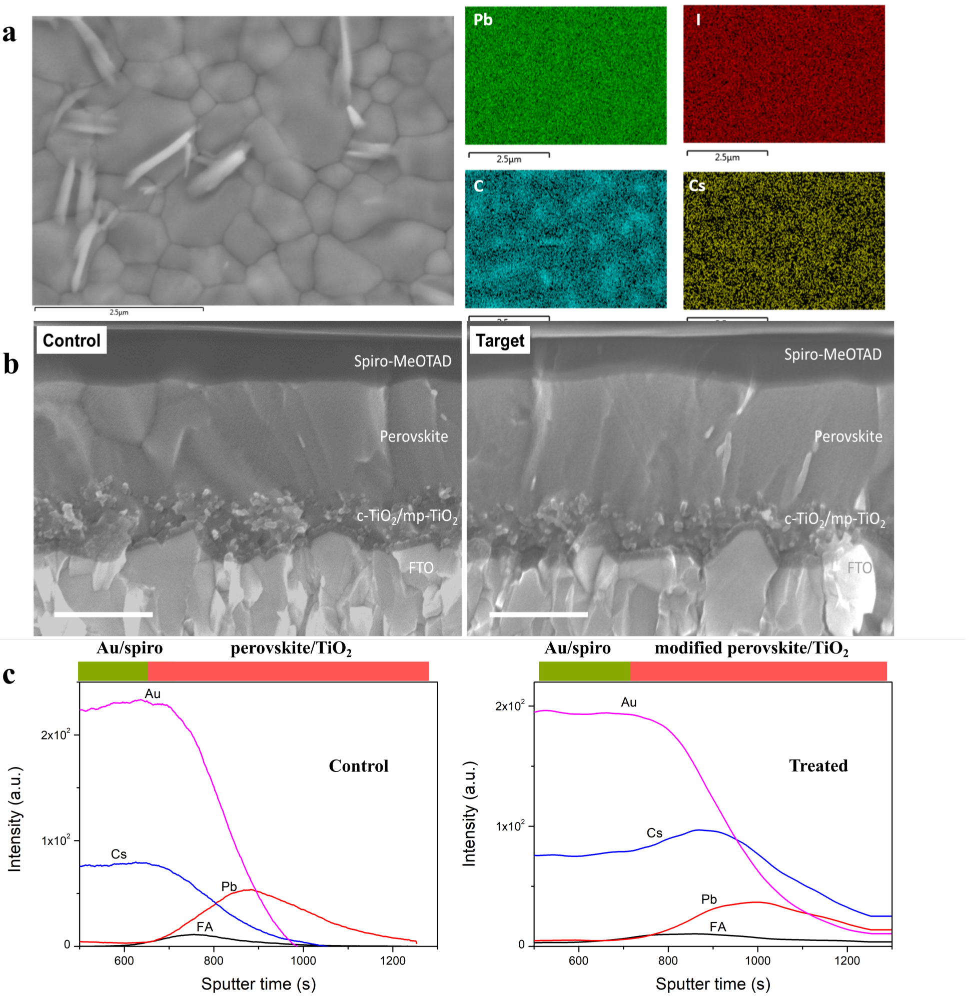

Fig. S6. Element distribution in perovskite film and the relevant devices. **a**, EDS mapping of perovskite film treated with CsI-DB21C7. **b**, Cross-sectional SEM images and of PSCs without (left) and with (right) CsI-DB21C7 treatment. The scale bar is 500 nm. **c**, TOF-SIMS spectra of PSCs without (left) and with (right) CsI-DB21C7 treatment.

Supplementary Note 1. Additional evidence for the infusion of Cs^+ ions

To further evidence the infusion of Cs^+ ions, XPS depth profiles were measured (Fig. S4c-d). After the treatment, the Cs 3*d* level signals could still be detected after sputtering of approximately 484 ± 49 nm (13th sputtering layer) into the perovskite film (thickness of 820 ± 80 nm). We estimate the Cs penetration length by combing the I 3*d* XPS depth profiles (Fig. S4c) and the cross-sectional SEM image of the target perovskite film (Fig. S6b). To corroborate this observation, we also performed time-of-flight secondary ion mass spectrometry (TOF-SIMS) and monitored compositional changes in the F-doped SnO_2 (FTO)/TiO_2/perovskite/2,2',7,7'-tetrakis[*N*,*N*-di(4-methoxyphenyl)amino]-9,9'-spirobifluorene (spiro-OMeTAD)/Au device architecture (Fig. S6c). This technique allows for depth profilng of atomic, as well as molecular A-site constituents, throughout the device stack[2]. The treated devices show a sharp increase in Cs concentration at the perovskite/spiro-OMeTAD interface, which then decreases deeper into the bulk of the perovskite film. Therefore, TOF-SIMS results are in accordance with the XPS, revealing that there is a gradient in the level of Cs doping (illustrated in Fig. 1b) in the films.

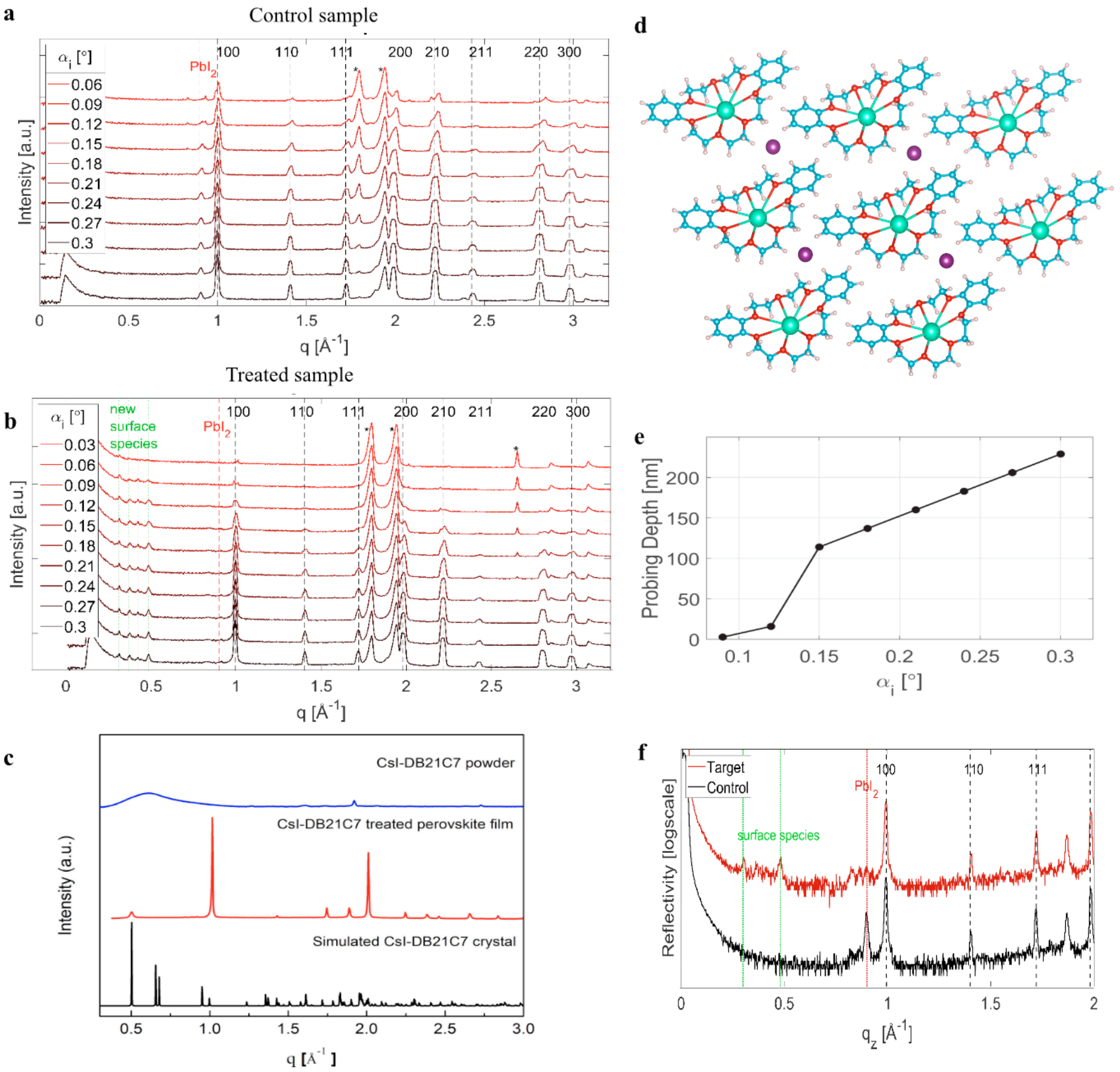

Fig. S7. Structural characterizations of perovskite film without and with CsI-DB21C7 treatment. a, b, Radial intensities of reciprocal space maps obtained from GIWAXS on control and treated perovskite films for different angles of incidence. **c**, Powder X-ray diffraction patterns of the simulated CsI-DB21C7 crystal, the CsI-DB21C7 treated perovskite film and CsI-DB21C7 powder. **d**, Visualization of the DFT optimized crystal structure of CsI-DB21C7. Color coding of species: Cesium-blue green, carbon-light blue, iodide-violet, oxygen-red and hydrogen-white. **e**, Probing depth vs incidence angle, estimated from calculation of the critical angle $\alpha_c \approx 0.12°$. **f,** XRR scans under ambient conditions.

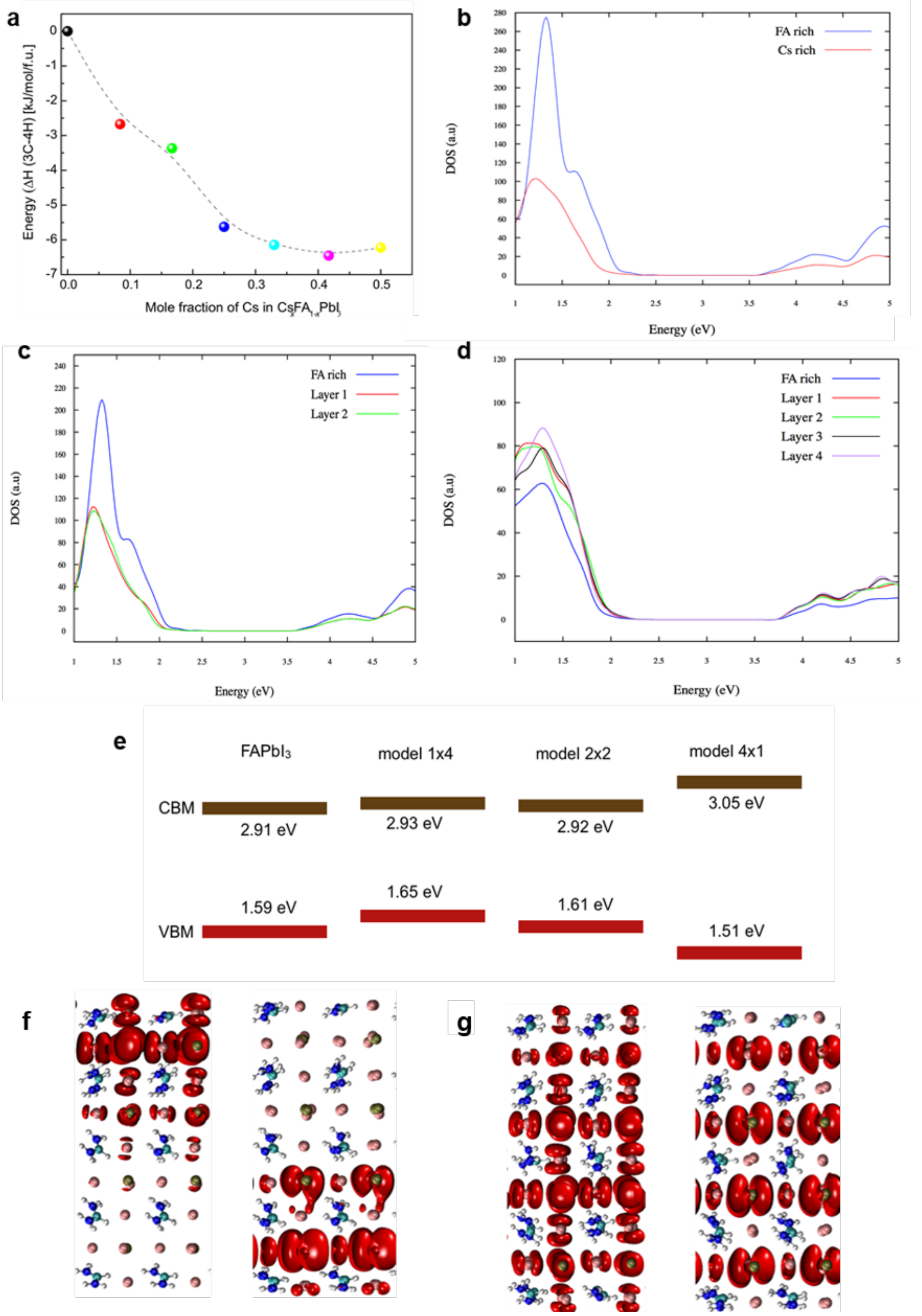

Fig. S8. Phase transition enthalpy and band structure of gradient Cs-doped $FAPbI_3$. a, The difference between the DFT-D3 energies of the 4H and pseudo-cubic phases of mixed cation $Cs_xFA_{1-x}PbI_3$ with different cesium concentrations. **b–d**,The projected density of states (PDOS) of 1×4 (b), 2×2 (c) and 4×1(d) models of Cs-doped $FAPbI_3$ structures. **e**, Valence band maximum (VBM) and conduction band minimum (CBM) corresponding to different models of Cs-doped $FAPbI_3$. **f–g,** DFT calculations of the Cs^+-treated $FAPbI_3$ containing a FA^+ vacancy. Visualized isosurfaces of the band edges orbitals (VBM on the left and CBM on the right) of FAI-terminated slabs of $FAPbI_3$, containing a FA^+ vacancy without (f) and with Cs^+-treatment (g).

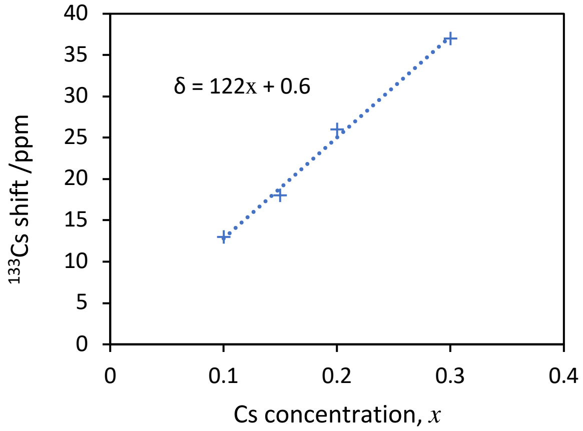

Fig. S9. Dependence of the [133]Cs chemical shift on the Cs concentration, x, in $Cs_xFA_{1-x}PbI_3$. Data taken from Kubicki et al.[3]

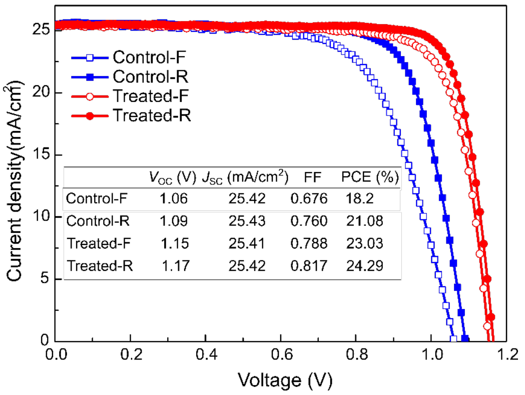

Fig. S10. Champion photovoltaic device performances. *J-V* curves of the champion $(FAPbI_3)_{1-x}(MAPbBr_3)_x$-based perovskite solar cells under reverse and forward scans are shown in Fig. 4b.

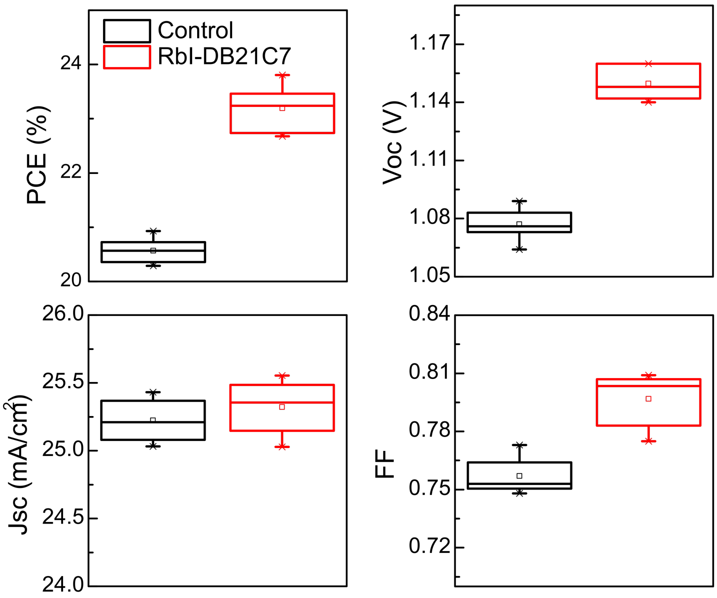

Fig. S11. Photovoltaic metrics of the $(FAPbI_3)_{1-x}(MAPbBr_3)_x$-based PSCs without and with RbI-DB21C7 treatments.

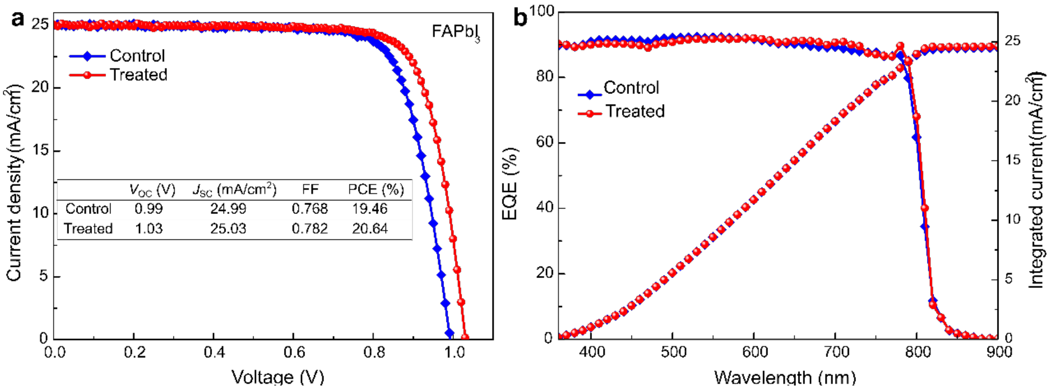

Fig. S12. (a) *J-V* curves and (b) IPCE spectra of typical pure (Br/MA free) $FAPbI_3$-based PSCs without and with CsI-DB21C7 treatment.

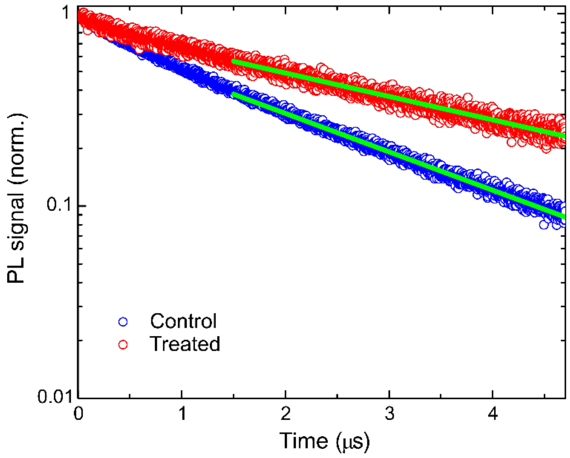

Fig. S13. TRPL spectra of perovskite films on glass substrate. Green lines in the 1.5–to 4.5 μs domain indicate curve fitting.

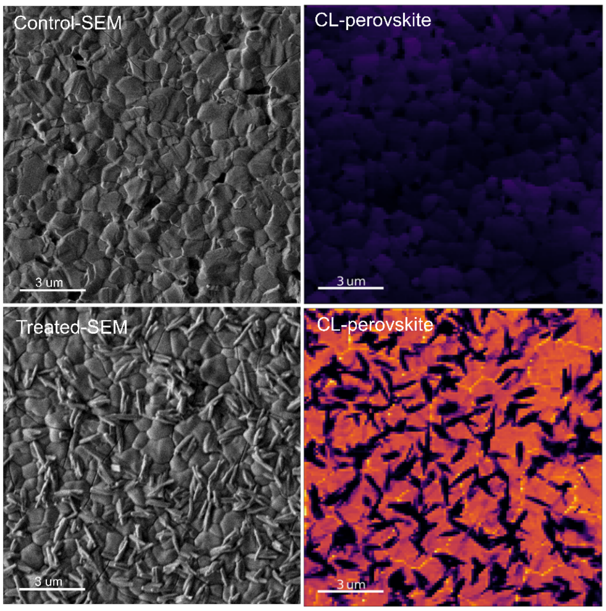

Fig. S14. Scanning electron microscopy (SEM) coupled-cathodoluminescence (CL) maps of the control (up) and treated (down) perovskite films on the FTO (1.1 mm) substrates. The spatial distribution of emission from 800 to 850 nm reveals perovskite in the films. The black needle-like species arises from the CsI-DB21C7 complex.

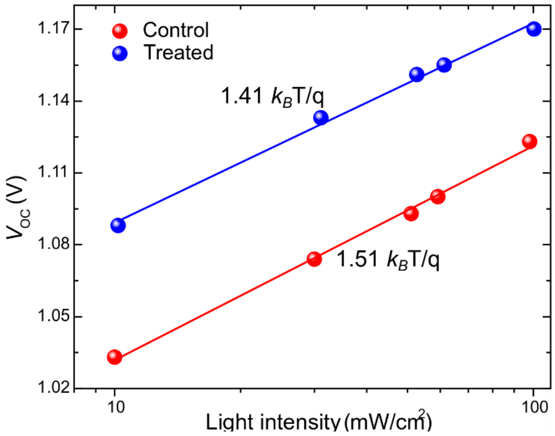

Fig. S15. V_{OC} light-intensity dependence. q: electron charge, k_B: Boltzmann constant, T: kelvin temperature.

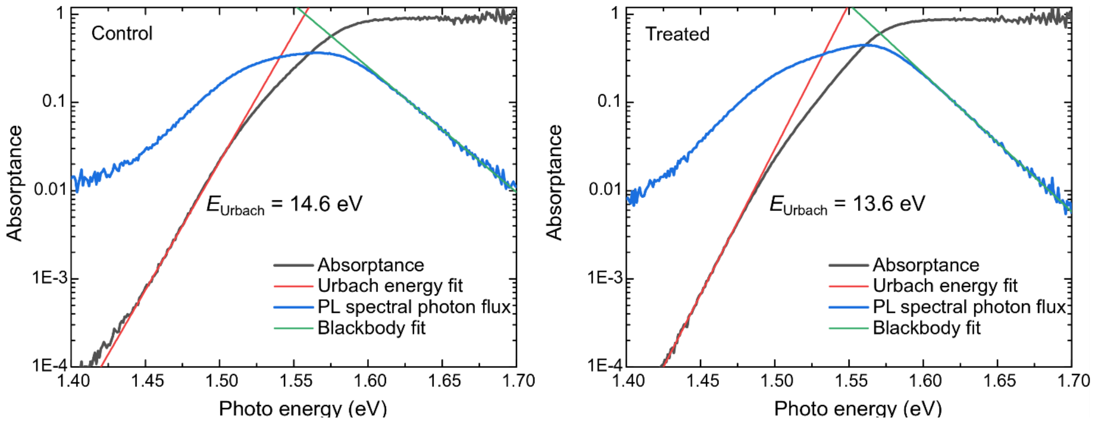

Fig. S16. Analysis of photoluminescence spectral photon flux for (c) control device and (d) treated device. Following the method detailed in the literature[4].

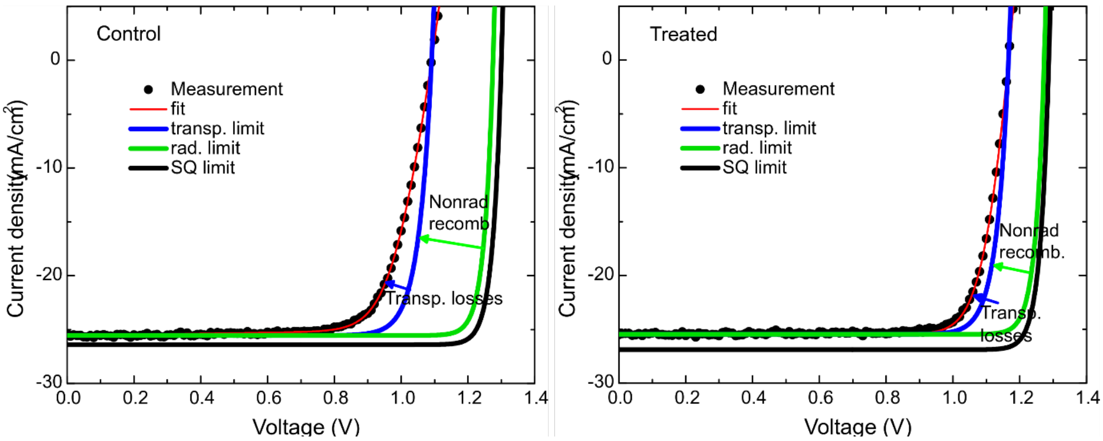

Fig. S17. J-V fits of control and treated devices, respectively, allowing for the breakdown of losses summarized in main Fig. 4d, following the procedure presented in the literature[5].

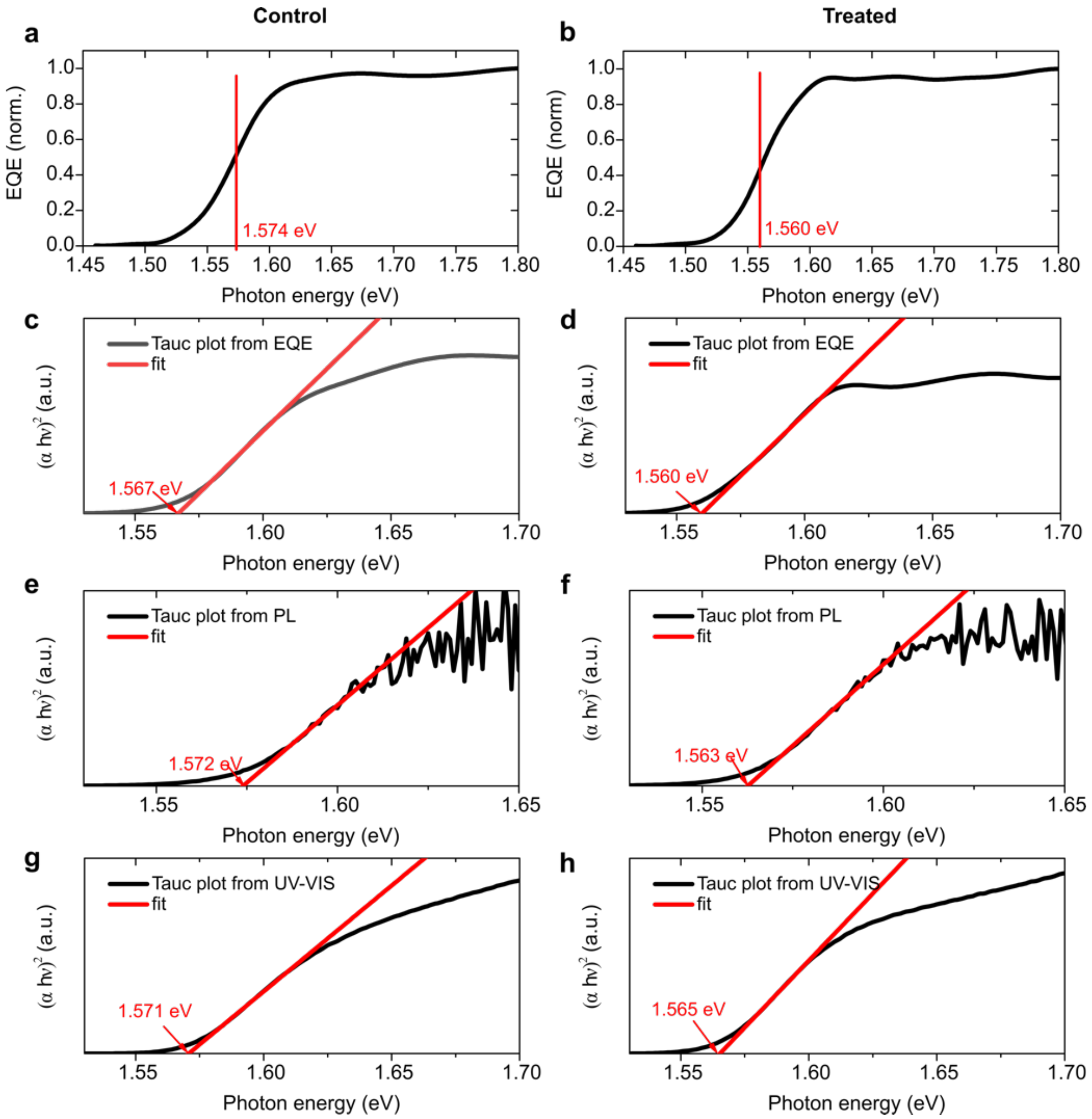

Fig. S18. Bandgap calculated with different techniques for control (a, c, e, g) and the treated film (b, d, f, h). The Tauc plots in e) and f) are calculated from the absorptance spectra derived from the PL spectra in Fig. 4c.

Supplementary Note 2. Analysis of devices' diode characteristics

The origin of the performance improvement is further analyzed by investigating the devices' diode characteristics. First, we evaluated the ideality factor n by measuring the dependence of V_{OC} on the incident light intensity (Fig. S15). Upon surface treatment, the ideality factor decreases from 1.51 to 1.41, indicative of suppressed non-radiative recombination channels[4]. Accordingly, we fit the J-V curves of the champion control and target devices to a single-diode equivalent circuit model[6] (Fig. S17 and Table S3). Following the analysis proposed in the literature[5], the losses can be broken down according to their origin (Fig. 4d). First, the Shockley-Queisser limit is calculated from the bandgap which we determined from the inflection point of the EQE spectrum[7]. For the control device we obtain a bandgap of 1.574 eV and for the target device 1.560 eV (the details of the bandgap determination and the comparison with other methods can be found in Table S4 and Fig. S18). These bandgap values are essentially the same and the small difference can be explained by the statistical compositional batch-to-batch fluctuations. Note that for the treated film this bandgap is for the part of the film that has the same composition as the control film, since it has the lowest bandgap, determining the low energy tail of the EQE and absorptance spectra. Next, the radiative limit J-V curve is calculated by using the absorptance spectra derived from the PL spectra (Fig. S16) and the J_{sc} of the champion cells, again assuming an ideal diode behavior (n = 1; with series and shunt resistances of R_{series} = 0 and R_{shunt} = ∞, respectively). The performance losses related to the radiative limit with respect to the Shockley-Queisser limit account for 5.1% for the control and 6.4% for the treated film, mainly stemming from J_{SC} losses due to non-ideal absorption. The contribution of non-radiative bulk and interface recombination is further evaluated by calculating the transport limit using the measured V_{OC} and ideality factor, while maintaining the J_{SC} and ideal resistances. Thereafter, the losses due to charge transport are obtained from the measured J-V curve. Consistent with the previous findings, a significant improvement in non-radiative losses is observed, decreasing from 20.2% in the control device to 12.6% in the target device, confirming the role of the reduction of non-radiative recombination as the main driver for performance improvement in our study. A notable improvement in transport losses (10.7% to 5.2%) is also observed, arising from decreases in series resistance and ideality factor, which are both traced to the suppression of interfacial defects or barriers.

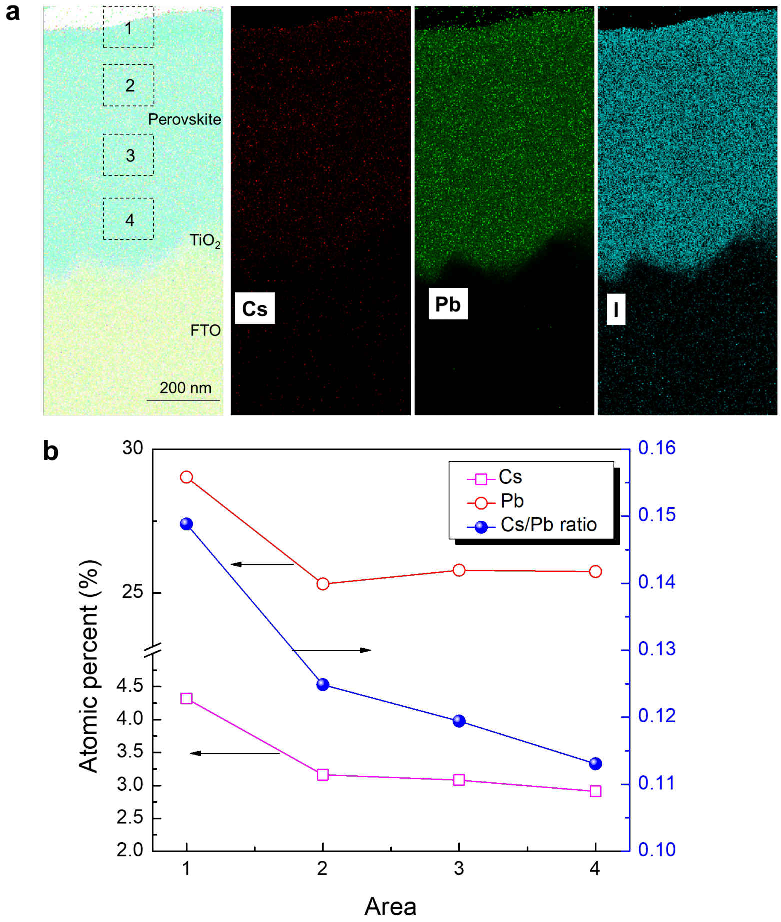

Fig. S19. Elemental mapping of the perovskite device before and after aging at elevated temperatures.

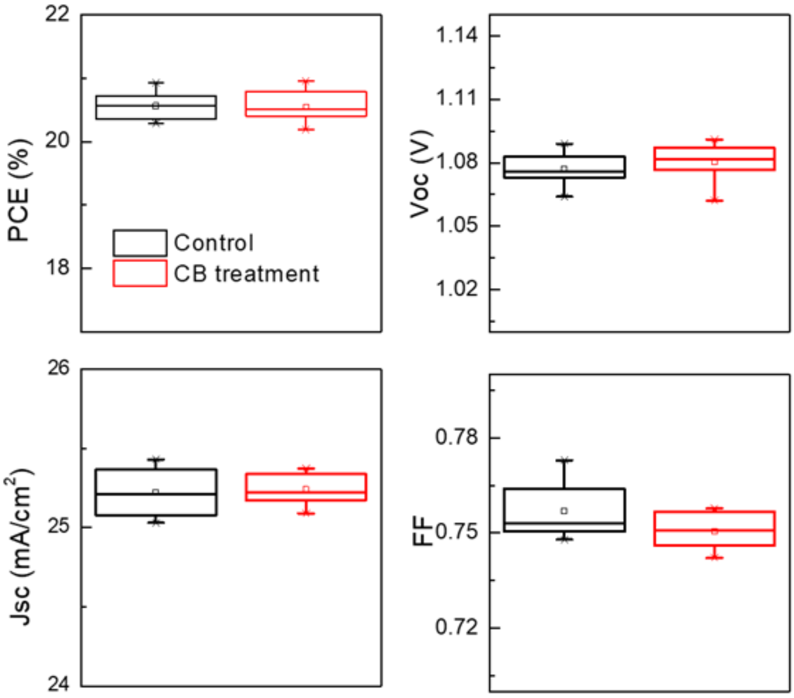

Fig. S20. Comparison of the control and chlorobenzene (CB)-treated perovskite solar cells excluding the effects of the antisolvent on the performance.

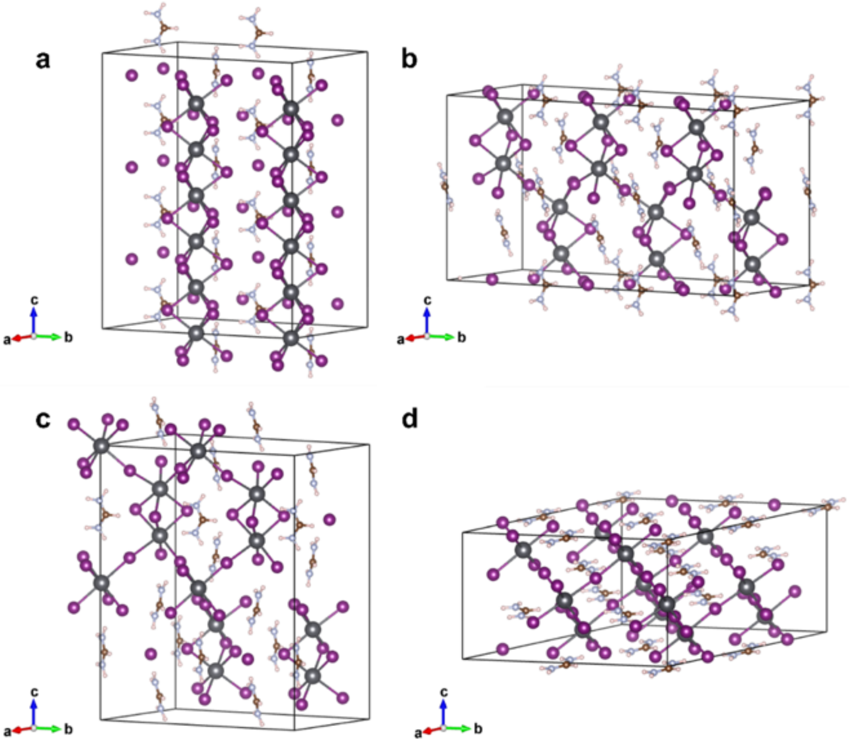

Fig. S21. Polytypes of $FAPbI_3$. (**a**) 2H, (**b**) 4H, and (**c**) 6H polytype. (**d**) Cubic-phase (3C polytype) of $FAPbI_3$. All the atoms are represented with ball and sticks configurations. Lead atoms are depicted with grey color, iodide with magenta color, carbon with dark brown color, nitrogen with light blue and hydrogen atoms with white spheres.

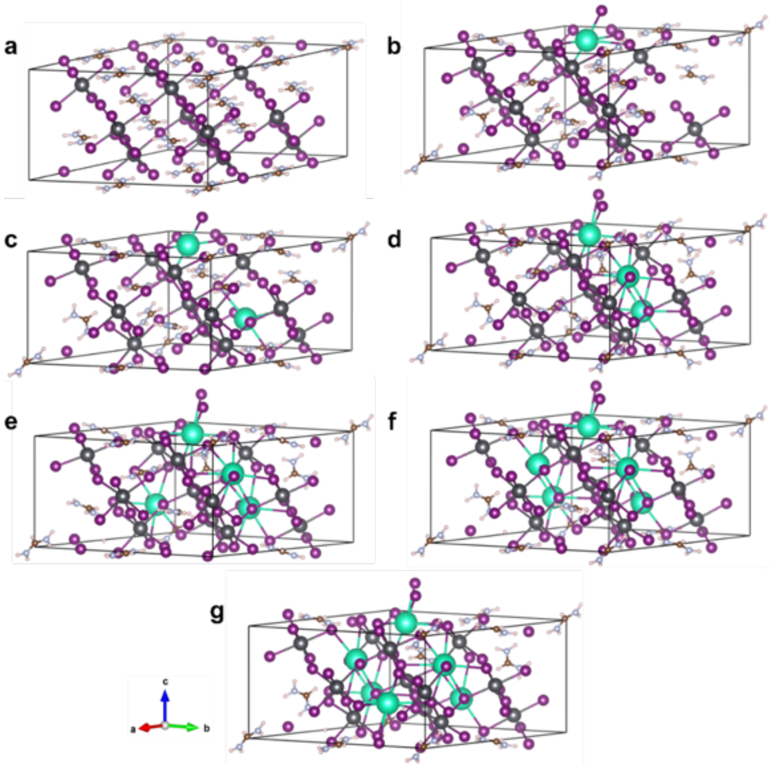

Fig. S22. Cs-doped cubic phase of $FAPbI_3$. (a-g) Cubic-phase of $FAPbI_3$ doped with the increasing amount of Cs by replacing FA cations. All the atoms are represented with ball and sticks configurations. Lead atoms are depicted with grey color, iodide with magenta color, cesium with bluish green color, carbon with dark brown color, nitrogen with light blue and hydrogen atoms with white spheres.

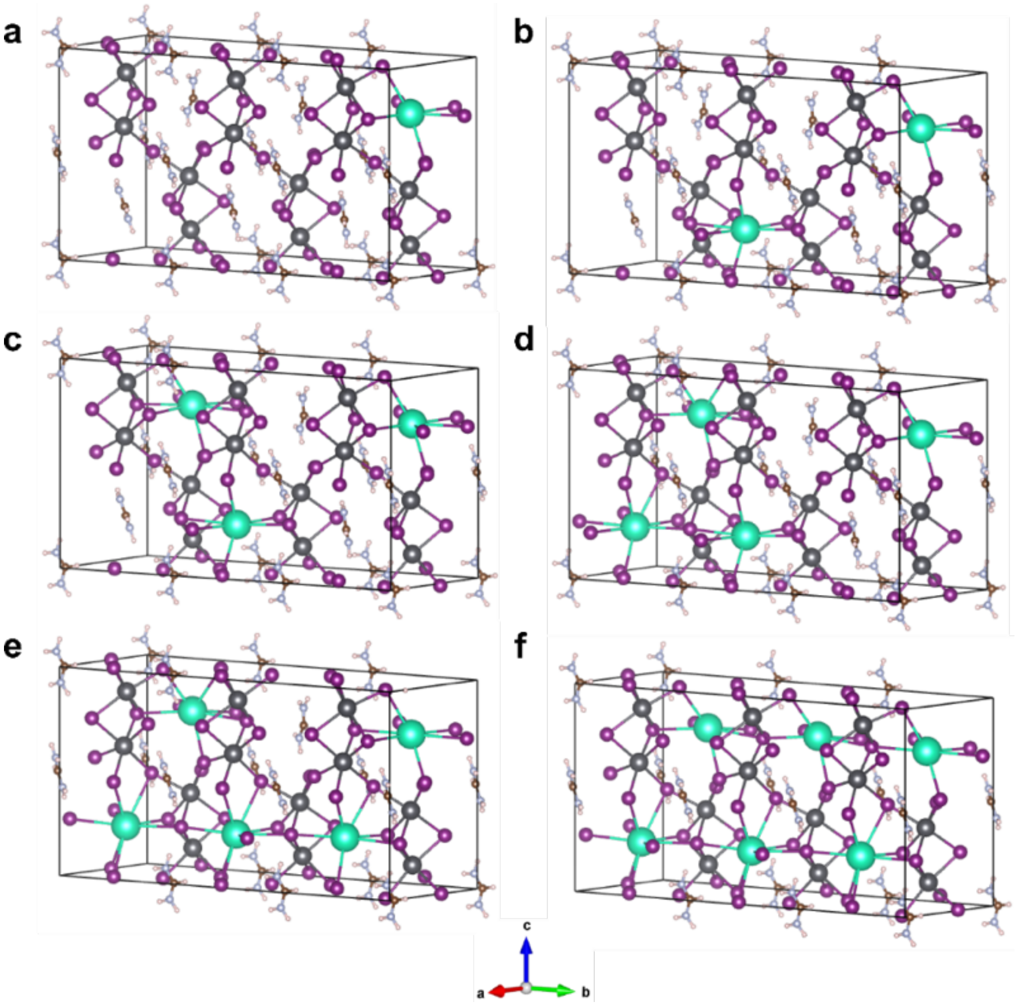

Fig. S23. Cesium doped 4H polytype of $FAPbI_3$. a-f, 4H-polytype of $FAPbI_3$ doped with the increasing amount of Cs by replacing FA molecules. All the atoms are represented with ball and sticks configurations. Lead atoms are depicted with grey color, iodide with magenta color, cesium with bluish green color, carbon with dark brown color, nitrogen with light blue and hydrogen atoms with white spheres.

Table S1. Summary of complexation energies of three possible reactions CsI-DB21C7 involved.

Reaction	Complexation Energy (eV)
DB21C7 + $Cs^+ \rightarrow$ DB21C7-Cs^+	- 2.22
DB21C7+ $FA^+ \rightarrow$ DB21C7-FA^+	- 1.68
DB21C7+ $Pb^{2+} \rightarrow$ DB21C7-Pb^{2+}	- 8.93

Table S2. Summary of the parameters from fits to the PL spectral photon flux measurement in Fig. S16.

	E_{Urbach} (meV)	$J_{\mathrm{rad,0}}$ (mA/cm^2)	Rad. limit of V_{OC} (V)
Control	14.6	7.11e-21	1.275
Treated	13.6	7.55e-21	1.273

Table S3. Performance parameters (n_{id}: ideality factor, R_s: series resistance, R_{sh}: shunt resistance) for the different diode characteristics shown in Fig. S17.

a) Control	n_{id}	R_S (Ω.cm^2)	R_{Sh} (Ω.cm^2)	V_{OC} (V)	J_{SC} (mA/cm^2)	FF (%)	PCE (%)
SQ lim.	1	0	∞	1.3	26.4	90.4	31
Rad. lim.	1	0	∞	1.275	25.6	90.3	29.4
Transp. lim.	1.51	0	∞	1.091	25.6	85	23.7
Experiment	1.78	2.88	$2.26 \cdot 10^3$	1.091	25.6	75.9	21.2
**b) Treated**	n_{id}	R_S (Ω.cm^2)	R_{sh} (Ω.cm^2)	V_{OC} (V)	J_{SC} (mA/cm^2)	FF (%)	PCE (%)
SQ lim.	1	0	∞	1.287	26.9	90.3	31.3
Rad. lim.	1	0	∞	1.273	25.5	90.3	29.3
Transp. lim.	1.41	0	∞	1.166	25.5	86.5	25.7
Experiment	1.52	1.53	∞	1.166	25.5	81.9	24.3

Table S4. Summary of bandgap calculations. The values shown are from Fig. S18 and in case of PL maximum from Fig. 4c. Note that the values obtained from the different techniques are different as discussed in detail by Krückemeier *et al.* [7]. We follow their advice and use the inflection point of the EQE (a and b) as the bandgap in this work.

**Method**	**Control E_g (eV)**	**Treated E_g (eV)**
EQE inflection point	1.574	1.560
EQE Tauc plot	1.567	1.560
UV-Vis Tauc plot	1.571	1.565
PL Tauc plot	1.572	1.563
PL maximum	1.565	1.558

References

1 A Veldhuis, S. *et al.* Crown ethers enable room-temperature synthesis of $CsPbBr_3$ quantum dots for light-emitting diodes. *ACS Energy Lett.* **3,** 526–531 (2018).

2 Greco, A. *et al.* Kinetics of ion-exchange reactions in hybrid organic–inorganic perovskite thin films studied by in situ real-time X-ray scattering. *J. Phys. Chem. Lett.* **9,** 6750–6754 (2018).

3 Kubicki, D. J. *et al.* Phase Segregation in Cs-, Rb- and K-doped mixed-cation $(MA)_x(FA)_{1-x}PbI_3$ hybrid perovskites from solid-state NMR. *J. Am. Chem. Soc.* **139**, 14173–14180 (2017).

4 Tress, W. *et al.* Interpretation and evolution of open-circuit voltage, recombination, ideality factor and subgap defect states during reversible light-soaking and irreversible degradation of perovskite solar cells. *Energy Environ. Sci.* **11**, 151–165 (2018).

5 Stolterfoht, M. *et al.* How to quantify the efficiency potential of neat perovskite films: perovskite semiconductors with an implied efficiency exceeding 28%. *Adv. Mater.* 2000080 (2020).

6 Zhang, C., Zhang, J., Hao, Y., Lin, Z. & Zhu, C. A simple and efficient solar cell parameter extraction method from a single current-voltage curve. *J. Appl. Phys.* **110**, 064504 (2011).

7 Krückemeier, L., Rau, U., Stolterfoht, M. & Kirchartz, T. How to report record open-circuit voltages in lead-halide perovskite solar cells. *Adv. Energy Mater.* **10**, 1902573 (2020).
