# Supplementary material for: Multimodal host–guest complexation for efficient and stable perovskite photovoltaics
Source: Nat Commun. 2021 Jun 7;12:3383. doi: 10.1038/s41467-021-23566-2 (PMC8185086; doi:10.1038/s41467-021-23566-2)
Supplement: Supplementary file 2 — Solar Cells Reporting Summary [file 41467_2021_23566_MOESM2_ESM.pdf]

nature research

Corresponding author(s): Michael Grätzel

Solar Cells Reporting Summary

Nature Research wishes to improve the reproducibility of the work that we publish. This form is intended for publication with all accepted papers reporting the characterization of photovoltaic devices and provides structure for consistency and transparency in reporting. Some list items might not apply to an individual manuscript, but all fields must be completed for clarity.

For further information on Nature Research policies, including our data availability policy, see Authors & Referees.

Experimental design

Please check: are the following details reported in the manuscript?

1. Dimensions

Area of the tested solar cells

☒ Yes ☐ No

The device area was 0.25 cm2 (0.5cm x 0.5 cm), as described in the Methods (Photovoltaic performance measurements).

Explain why this information is not reported/not relevant.

Method used to determine the device area

☒ Yes ☐ No

Described in Methods (Photovoltaic performance measurements). A black metal mask with an area of 0.16 cm2 (0.4cm x 0.4 cm) is used to define the active area.

Explain why this information is not reported/not relevant.

2. Current-voltage characterization

Current density-voltage (J-V) plots in both forward and backward direction

☒ Yes ☐ No

The manuscript provides current density - voltage (JV) plots in both forward and backward direction (Supplementary Fig. 10).

Explain why this information is not reported/not relevant.

Voltage scan conditions
For instance: scan direction, speed, dwell times

☒ Yes ☐ No

Voltage scan conditions are reported in the manuscript and described in the Methods (Photovoltaic performance measurements).

Explain why this information is not reported/not relevant.

Test environment
For instance: characterization temperature, in air or in glove box

☒ Yes ☐ No

The current-voltage characteristics are obtained under ambient temperature and air conditions, as reported in the manuscript.

Explain why this information is not reported/not relevant.

Protocol for preconditioning of the device before its characterization

☒ Yes ☐ No

Current-voltage characteristics were recorded in accordance with the procedure reported in the manuscript and in the Methods. No specific protocol for preconditioning was applied.

Explain why this information is not reported/not relevant.

Stability of the J-V characteristic
Verified with time evolution of the maximum power point or with the photocurrent at maximum power point; see ref. 7 for details.

☒ Yes ☐ No

Maximum power point tracking was conducted, as reported in the manuscript (Fig. 4b).

Explain why this information is not reported/not relevant.

3. Hysteresis or any other unusual behaviour

Description of the unusual behaviour observed during the characterization

☒ Yes ☐ No

Very low hysteresis was observed upon altering the scan direction is reported in the manuscript (Supplementary Fig.10).

Explain why this information is not reported/not relevant.

Related experimental data

☒ Yes ☐ No

The manuscript provides current density - voltage (JV) plots in both forward and backward direction with related experimental data provided in the manuscript (Supplementary Fig. 10).

Explain why this information is not reported/not relevant.

4. Efficiency

External quantum efficiency (EQE) or incident photons to current efficiency (IPCE)

☒ Yes ☐ No

IPCE spectra were recorded, as reported in Supplementary Fig. 12b.

Explain why this information is not reported/not relevant.

A comparison between the integrated response under the standard reference spectrum and the response measure under the simulator

☒ Yes ☐ No

The integrated Jsc from IPCE spectra is consistent with the Jsc from JV measurements, as detailed in the manuscript (Supplementary Fig. 12).

Explain why this information is not reported/not relevant.

For tandem solar cells, the bias illumination and bias voltage used for each subcell

☐ Yes — *State where this information can be found in the text.*
☒ No — Not applicable as no tandem solar cells are reported in this work.

5. Calibration

Light source and reference cell or sensor used for the characterization

☒ Yes — The light source was a 300-W Xenon lamp (Oriel) equipped with a SchottK113 Tempax sunlight filter (Praezisions Glas & OptikGmbH) to match the emission spectrum of the lamp to the AM1.5G standard. Before each measurement, the exact light intensity was determined using a calibrated Si reference diode equipped with an infrared cut-off filter (KG-3, Schott), as detailed in the Methods (Photovoltaic performance measurements).
☐ No — *Explain why this information is not reported/not relevant.*

Confirmation that the reference cell was calibrated and certified

☒ Yes — The Si reference cell was calibrated and certified by Newport Corporation PV Lab, Bozeman, MT, USA, as detailed in the Methods.
☐ No — *Explain why this information is not reported/not relevant.*

Calculation of spectral mismatch between the reference cell and the devices under test

☒ Yes — The spectral mismatch between our simulator and the AM 1.5 solar source was insignificant as the integrated current densities estimated from the IPCE spectra were in good agreement with the values obtained from the current density - voltage (J-V) curves as detailed in the manuscript. Spectra mismatch factor of 1 was used.
☐ No — *Explain why this information is not reported/not relevant.*

6. Mask/aperture

Size of the mask/aperture used during testing

☒ Yes — All measurements were conducted using a non-reflective metal mask with an aperture area of 0.16 cm2 to cover part of the active area of the device and avoid stray light capturing by our device, as detailed in the Methods (Photovoltaic performance measurements).
☐ No — *Explain why this information is not reported/not relevant.*

Variation of the measured short-circuit current density with the mask/aperture area

☐ Yes — *State where this information can be found in the text.*
☒ No — We haven't measured the cells with apertures of different sizes.

7. Performance certification

Identity of the independent certification laboratory that confirmed the photovoltaic performance

☐ Yes — *State where this information can be found in the text.*
☒ No — The results have not been certified yet by an independent accredited laboratory. However, our devices of the same aperture area (0.16 cm2) have previously been certified by an independent accredited laboratory (Newport Corporation PV Lab, Bozeman, MT, USA), as reported in our previous work (Science 2016, 353, 58–62).

A copy of any certificate(s)
Provide in Supplementary Information

☐ Yes — *State where this information can be found in the text.*
☒ No — Not applicable as we have not yet certified our devices by an independent accredited laboratory.

8. Statistics

Number of solar cells tested

☒ Yes — At least 20 devices for each condition were tested, as reported (Fig. 4a).
☐ No — *Explain why this information is not reported/not relevant.*

Statistical analysis of the device performance

☒ Yes — Histograms of efficiency for the devices are reported (Fig 4a).
☐ No — *Explain why this information is not reported/not relevant.*

9. Long-term stability analysis

Type of analysis, bias conditions and environmental conditions
For instance: illumination type, temperature, atmosphere humidity, encapsulation method, preconditioning temperature

☒ Yes — The long-term stability analysis is detailed in the manuscript with respect to the type of analysis, illumination, bias, and environmental conditions (involving temperature and atmosphere humidity), as well as the exclusion of encapsulation, which is detailed in the manuscript. The operational stability was evaluated by maximum power point tracking measured with the unencapsulated device under full solar illumination (AM 1.5 G, 100 mW/cm2 in N2, 25 °C).
☐ No — *Explain why this information is not reported/not relevant.*
