# Supplementary figures and images for: Multimodal host–guest complexation for efficient and stable perovskite photovoltaics
Source: Nat Commun. 2021 Jun 7;12:3383. doi: 10.1038/s41467-021-23566-2 (PMC8185086; doi:10.1038/s41467-021-23566-2)

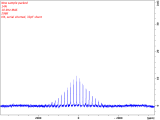

Supplement: Supplementary file 3 — Source Data [file 41467_2021_23566_MOESM3_ESM.zip › Source data/ssNMR-data/14N/3/pdata/1/thumb.png]
